# Optimal dose and type of exercise to improve cognitive function in patients with mild cognitive impairment: a systematic review and network meta-analysis of RCTs

**DOI:** 10.3389/fpsyt.2024.1436499

**Published:** 2024-09-12

**Authors:** Yingying Yu, Junjie Wang, Jian Xu

**Affiliations:** School of Sport and Health Sciences, Dalian University of Technology, Dalian, China

**Keywords:** cognitive function, mild cognitive impairment, exercise, dose-response relationship, network meta-analysis

## Abstract

**Background:**

Mild cognitive impairment (MCI) represents a prodromal stage of dementia, characterized by cognitive decline exceeding that expected with normal aging. Exercise interventions have emerged as a promising approach to counter functional decline and enhance cognitive function in the elderly MCI population. However, the optimal exercise modalities and dosage (dose-response relationship) are understudied.

**Objective:**

It aims to determine the most effective exercise modality for MCI patients by optimizing the dose-response relationship to ensure sufficient intensity to induce positive neurological adaptations.

**Methods:**

A systematic search of electronic databases, including PubMed, Embase, Scopus, Web of Science, and Cochrane Central Register of Controlled Trials was conducted from inception to April 15, 2024. Studies evaluating the efficacy of exercise interventions in MCI participants were included. Primary outcomes of interest are global cognition and executive function. Random-effects models will be utilized for both pairwise and network meta-analysis.

**Results:**

Following the application of specific inclusion and exclusion criteria, a total of 42 articles, encompassing 2832 participants, were chosen for inclusion in a network meta-analysis. The findings revealed that multi-component exercise demonstrated superior efficacy in mitigating the deterioration of global cognition, as evidenced by standard mean differences (SMDs) of 1.09 (95% CI: 0.68 to 1.51) compared to passive controls. Additionally, multi-component exercise exhibited a significant impact on executive function, with SMDs of 2.50 (95% CI: 0.88 to 4.12) when contrasted with passive controls. Our research has demonstrated that sessions lasting 30 minutes, occurring 3-4 times per week, with interventions lasting 12-24 weeks and an intensity of 60-85% of maximum heart rate, yield higher effect sizes in improving global cognition. However, sessions lasting 30-61 minutes, with interventions lasting 25 weeks or longer, show greater effectiveness in enhancing executive function.

**Conclusion:**

A network meta-analysis identified multi-component exercise as the most effective intervention for improving global cognitive and executive function in patients with mild cognitive impairment. Notably, moderate-intensity exercise performed at least three times weekly appears beneficial, with evidence suggesting shorter sessions and higher frequencies may optimize cognitive outcomes.

**Systematic Review Registration:**

https://www.crd.york.ac.uk/PROSPERO, identifier CRD42024534922.

## 1 Introduction

### 1.1 Elderly people with MCI

As the demographic shift towards an older population continues (1), there is a significant rise in the number of elderly individuals experiencing cognitive disorders such as Alzheimer’s disease, dementia, and mild cognitive impairment (2). It is also reported that the number of older adults with cognitive problems reached one-third, and approximately one-fifth older adults suffer from mild cognitive impairment, and another one-seventh suffer from dementia (3). This trend has emerged as a pressing public health concern, prompting increased focus from healthcare providers, researchers, and policymakers (4–6). Meanwhile we learned that a heavy social and economic burden is expected to result from cognitive impairments associated with aging, and will be US $2.54 trillion in 2030 (7). The mild cognitive impairment, in particular, is recognized as a transitional phase between normal age-related cognitive decline and the more severe symptoms associated with dementia (8), including memory loss, language difficulties, and impaired judgement (9, 10). MCI is estimated to be prevalent among people over 60 years old by about 15% (11).Individuals with mild cognitive impairment and their family members may recognize alterations in cognitive function, though these changes may not be severe enough to significantly impact daily functioning or disrupt typical activities (12, 13). MCI is associated with an elevated risk of developing dementia, particularly Alzheimer’s disease or other neurological disorders (14). However, the progression of MCI can vary among individuals, with some experiencing stability, deterioration, or even improvement in cognitive abilities (15). While individuals with MCI face an increased risk of developing dementia, it is not a certainty (16). Studies show that 10 to 15% of people with MCI go on to develop dementia within a year (17). Dementia affects about 1% to 3% of older adults each year (15, 18).

### 1.2 Exercise improves MCI

Recent research suggests that exercise may enhance cognitive function in individuals with mild cognitive impairment (19). Studies have shown that exercise interventions can potentially reverse functional decline and improve cognitive abilities in elderly patients with MCI (20, 21). It has been shown in RCTs that exercise can enhance cognitive functioning in older adults, including global cognition and executive function (22–24). Additionally, neuroimaging studies have demonstrated that exercise can positively impact brain structure and functional connectivity by enhancing levels of growth factors like brain-derived neurotrophic factor (BDNF) (25, 26). Numerous studies have demonstrated that various exercise modalities may exert their beneficial effects through distinct molecular mechanisms (27). Consequently, it is imperative to ascertain the most efficacious types of exercise for enhancing cognitive function in mild cognitive impairment individuals (28). NMA offer a means of comparing interventions for a specific condition, providing quantitative evaluations and rankings of their efficacy (29). Thus, enabling the identification of the optimal exercise regimen for patients with MCI.

### 1.3 Exercise dose-response relationship

Currently, numerous studies have examined the impact of exercise on enhancing cognitive function in individuals with MCI. However, there is a lack of definitive research analyzing and discussing the optimal exercise dosage with ME (30).The exercise dose-response relationship is a significant factor in enhancing cognitive function in MCI individuals (31). Achieving an optimal balance is essential to maximize benefits while minimizing the risk of fatigue or injury (32, 33). Components of dose-related effects in exercise therapy include parameters such as training intensity, frequency, and duration (34). Exercise dose parameters have been linked to enhanced fitness levels, potentially impacting cognitive function through the promotion of brain plasticity (35). The purpose of this review is to find out the most effective dose parameters for enhancing cognitive function, specifically global cognition and executive function in mild cognitive impairment individuals (36). The study quantified the dose relationship of optimal exercise on global cognition and executive function using advanced methods (37). Improving the quality of life of elderly people with cognitive impairment by improving their brain health (38).

To sum up, there are two main issues in this paper, one is which types of exercise are most effective for patients with mild cognitive impairment, and the other is which parameters define the optimal exercise dose.

## 2 Methods

According to the PRISMA guidelines, this systematic review was preregistered with a meta-analysis PROSPERO reference number (CRD42024534922) (39). To accomplish this program, authors followed the Cochrane Handbook for Systematic Reviews of Interventions.

### 2.1 Data sources

A systematic search was conducted on the PubMed, Embase, Scopus, Web of Science, and Cochrane Central Register of Controlled Trials databases from their inception to April 15, 2024. The search strategy is detailed in **Appendix 1**. In addition to screening titles/abstracts and full-text articles independently and in duplicate, discrepancies were resolved through discussion with a third author, XJ.

### 2.2 Inclusion and study selection

For the network meta-analysis (NMA) and review to be included, studies had to meet the following criteria: (1) the participants had to have been diagnosed with MCI (2) age 60 years (3) RCTs (4) were written in English (5) experimental group used various type of exercise intervention (6) control group may not receive exercise intervention (usual care, health education), or other exercise intervention methods (7) outcome including global cognition and executive function and above. The exclusion criteria are as follows: (1) cognitive impairment patients with Parkinson’s disease, dementia, or psychiatric illness (due to pathological changes accompanying exercise that may have confounded its effects) (2) non-RCTs (3) lack of extractable outcomes (4) non-English peer-reviewed full text (5) conference abstracts (6) reviews of the literature full text.

### 2.3 Data extraction

The data extraction process was conducted independently by two reviewers (YY and WJ), with disagreements resolved through discussion to achieve consensus. In cases where disagreements persisted, a third reviewer (XJ) made the final decision. The extracted data included study and participant characteristics, exercise intervention measures of experimental group and control group, exercise dose parameters (such as frequency, duration per session, length of intervention), and outcomes (statistical data at the endpoint of the intervention) as outlined in the **Table 1**. Missing data were addressed by contacting the author via email.

**Table 1:** Study characteristics.

study	Participants (exercise vs control)				interventions					Outcomes	
	Baseline	Sample size	Age	Female/male	Type	Int	Dur	Freq	Len	Comparator	(GC=global cognition; Ex=executive function)
Scherder, E. J. 2005 (59)	MCI	15 vs 15	84 ± 6.38 vs 86 ± 5.05	13/2 vs 14/1	self-paced slow walking (AE)		30	3	6	social visit and normal activity	Ex (TMT A+B) 339.67 ± 207.41vs 253.73 ± 150.03
Varela, S.2012 (60)	MCI	17 vs 16 vs 15	79.24 ± 10.07 vs 76.44 ± 11.38 vs 79.40 ± 6.72	27/21	(1) aerobic exercise (2) aerobic exercise	(1) 40% of HRR (2) 60% of HRR	30	3	13	recreational activities	GC (MMSE) 20.40 ± 4.15 vs 20.98 ± 5.4 vs 19.53 ± 5.5
Lam, L. C.2012 (61)	MCI	92 vs 169	77.2 ± 6.3 vs 78.3 ± 6.6	125/46 vs 172/46	Taichi (MBE)		30	3	52	Stretching and toning	GC (MMSE) 25.4 ± 3.3 vs 24.2 ± 3.4 Ex (TMT-B)-102.3 ± 51.6 vs -125.2 ± 69.5
Suzuki, T.2012 (62)	aMCI	25 vs 25	75.3 ± 7.5 vs 76.8 ± 6.8	13/12 vs 14/11	aerobic, strength, balance retraining (ME)	60%HRmax, low intensity	90	2	52	education control	GC (MMSE) 26.33 ± 3.1 vs 26.16 ± 3.15 Ex (SCWT-III) 39.39 ± 19.45 vs 36.93 ± 19.74
Davis JC 2013 (63)	MCI	28 vs30 vs 28	74.1 ± 3.6 vs 75.5 ± 3.5 vs 75.0 ± 3.7	NA	(1) resistance training (2) aerobic training	(1) high intensity (2) 40%-60% HRR	60	2	26	balance and tone	Ex (Stroop Test) -44.61 ± 25.8 vs -48.27 ± 31.3 vs -54.69 ± 31.3
Fiatarone Singh, M. A.2014 (64)	MCI	22 vs 27	NA	NA	Progressive resistance training	high intensity, for most major muscle groups	75	2	24	Placebo	GC(ADAs-cog) -5.56 ± 3.06 vs -7.14 ± 2.94 Ex (WAIS-III Similarities) 22.35 ± 4.601 vs 19.02 ± 5.331
Wei, XH. 2014 (65)	MCI	30 vs 30	66.73 ± 5.48 vs 65.27 ± 4.63	9/21 vs 19/11	Handball training program (ME)	60%HRmax, low intensity	30	5	26	usual care	GC(MMSE) 25.53 ± 0.82 vs 24.67 ± 1.42
Lü, J. 2015 (66)	MCI	22 vs 23	69.00 ± 3.83 vs 70.43 ± 5.53	16/6 vs 16/7	dumbbell-training (RE)		60	3	12	usual care	GC (ADAS-Cog) -7.85 ± 2.80 vs -12.87 ± 4.80 Ex (TMT-B) -95.45 ± 45.00 vs -117.85 ± 54.01
Phoemsapthawee, J.2016 (67)	MCI	12 vs 12	65 to 87 years	All women	supervised ASE for 5 d·wk-1(AE)	23% VO2 peak Low intensity	30	5	12	usual care	GC(MMSE) 20.9 ± 5.1 vs 19.1 ± 4.8
Greblo Jurakic, Z. 2017 (68)	MCI	14 vs 14	70.4± 3.93 (Total)	all women	HUBER: balance and core resistance training (ME)	50-75% max voluntary contraction	30	3	8	Pilates:1h/session (MBE)	GC (MoCA) 25.79 ± 1.53 vs 24.29 ± 1.98 Ex (Visuospatial/executive) 3.50 ± 1.23 vs 2.57 ± 1.16
Kohanpour, M. A.2017 (69)	MCI	10 vs 10	60-70 (range)	all men	Aerobic running	75-85% HRmax	21-39	3	12	usual care	GC (MMSE) 24.4 ± 1.42 vs 24.2 ± 0.63
Lazarou, I.2017 (70)	aMCI	66vs 63	65.89 ± 10.76 VS 67.92 ± 9.47	53/13 vs 48/16	Dance (MBE)		60	2	43	usual care	GC(MMSE) 28.00 ± 2.39 VS 25.65 ± 3.27 Ex (ROCFT) 16.18 ± 6.02 vs 11.10 ± 6.50
Morris, J. K. 2017 (71)	MCI or dementia	39 vs 37	74.4 ± 6.7 vs 71.4 ± 8.4	18/21 vs 21/16	aerobic exercise	60 ± 75% HR reserve	30-50	3-5	26	stretching and toning	Ex(composite) -1.20 ± 0.90 vs -1.33 ± 0.97
Shimizu, N. 2018 (72)	MCI	30 vs 9	74.90 ± 4.29 vs 73.33 ± 7.31	28/6 vs 10/1	MMT: exercise; synchronization with music (MBE)		60	1	12	exercises; no music (ME)	Ex (FAB) -15.87 ± 1.66 vs -15.25 ± 1.16
Yoon DH 2017 (73)	MCI	14 vs 9 vs 7	75.0 ± 0.9 vs 76.0 ± 1.3 vs78.0 ± 1.0	NA	elastic exercise bands (RE)	HSPT: low tension LSST: high tension	60	2	12	balance and tone	GC(MMSE) 25.36 ± 1.78 vs 24.56 ± 3.21 vs 21.14 ± 1.57
Doi,2017 (74)	MCI	67 vs 67	75.7 ± 4.1) vs 76.0 ± 4.9	34/33 vs 31/36	Dance (MBE)		60	1	40	health education	GC(MMSE) 26.29 ± 2.6 vs 25.44 ± 2.3 Ex (TMT-B) -41.08 ± 11.2 vs -43.5 ± 11.8
Choi, W.2018 (75)	MCI	30 vs 30	74.90 ± 5.10 vs 74.23 ± 4.38	24/5 vs 25/5	ground kayak paddling (MBE)		60	2	6	home exercise	GC (MoCA) 25.13 ± 2.78 vs 21.46 ± 3.11
Combourieu DL. 2018 (76)	MCI	18 vs 14	77.1 ± 1.44 vs 79.2 ± 4	NA	aerobic training on bikes	moderate intensity	60	2	12	usual care	Ex (Stroop test) 30.94 ± 6.24vs 26.42 ± 6.53
Hong, 2018 (77)	MCI	10 vs 12	77.71 ± 3.40/78.33 ± 3.21 vs 75.11 ± 4.45/78.33 ± 5.50(F/M)	7/3 vs 9/3	resistance exercises	elastic band 15RM, 65%max	60	2	12	usual care	GC(MoCA) 21.70 ± 3.05 vs 20.50 ± 5.05 Ex (Stroop test) 13.40 ± 4.60 vs 12.25 ± 5.46
Sungkarat, S.2018 (78)	MCI	33 vs 33	68.3 ± 6.7 vs 67.5 ± 7.3	31/2 vs 26/7	Tai chi (MBE)		50	3	26	educational control	Ex (TMT) -76.2 ± 46.7 vs -101.1 ± 60.8
Zhu, Y. 2018 (79)	MCI	29 vs 31	70.3 ± 6.7 vs 69.0 ± 7.3	15/14 vs 21/10	Dance (MBE)	60-80%Hrmax moderate intensity	35	3	13	usual care	GC(MoCA) 24.7 ± 2.2vs 23.6 ± 1.8 Ex (TMT) -158 ± 49 VS -177 ± 48
Amjad, I.2019 (80)	MCI	21vs 19	58 ± 2 vs 60 ± 3	10/11 vs 9/10	stationary bicycle 40 min (AE)	60-80%HRmax	30	3	6	gentle movement	GC(MMSE) 26.353 ± 0.469 vs24.177± 0.849 Ex (TMT) -2.803 ± 0.282 vs -3.811 ± 0.205
Bademli, K. 2019 (81)	MCI	30 vs 30	72.24 ± 7.16 vs 70.67 ± 8.34	18/12 vs 17/13	Physical Activity Program: rhythmic exercises and free walking (ME)	3 to 6 metabolic equivalent Moderate intensity	80	4	20	usual care	GC(MMSE) 26.542 ± 1.84 vs 22.24 ± 1.15
Cardalda, I. M.2019 (82)	mild—moderate	25 vs 23 vs 29	85.54 ± 8.09 vs 83.76 ± 8.33 vs 85.17 ± 7.38	NA	(1) TG: strength-based physical exercise (RE) (2) MG calisthenics exercises (ME)	(1) resistance elastic bands (2) low intensity	60	2	12	Usual care (social activities)	GC(MMSE) 19.32 ± 7.10 vs 20.68 ± 7.68 vs 15.44 ± 7.55
Choi, W. 2019 (83)	MCI	30 vs 30	77.27 ± 4.37 vs 75.37 ± 3.97	25/5 vs 26/4	kayak paddling exercise with observing a video (MBE)		60	2	6	Home exercises	GC(MoCA) 23.22 ± 4.48 vs 20.12 ± 3.53
de Oliveira Silva, F. 2019 (84)	MCI	7 vs 12	71.85 ± 5.69 vs 78.20 ± 5.26	6/1vs5/7	Multimodal exercises: aerobic, strength, balance and flexibility	70-80%VO2max	60	2	13	usual care	GC(MMSE) 20.31± 4.68 vs 19.50 ± 5.37 Ex (Stroop test 3) 43.51 ± 52.71 vs 40.08 ± 74.27
Fonte, C.2019 (85)	MCI	7 VS 9	75 ± 5 vs 79 ± 3	3/4vs7/2	Multimodal exercises: cycling, walking, arm cranking, stretching	70%HRmax	90	3	26	usual care	Ex (TMT)-190.1 ± 30.6 vs -297.3 ± 71
Langoni, C. D. S. 2019 (86)	MCI	26 vs 26	72.6 ± 7.8 vs 71.9 ± 7.9	20/6 vs20/6	Multimodal exercises: strength and aerobic	60-75%HRmax	60	2	24	usual care	GC(MMSE) 25.0 ± 4.7 vs 20.4 ± 4.1
Law, L.L.F 2019 (87)	MCI	14 vs 16 vs 14	71.57 ± 7.43vs 77.94 ± 6.11vs 75.14 ± 8.53	10/4 vs 8/8 vs 9/5	(1) Functional task exercise (ME) (2) Exercise training (AE)	Low intensity	60	2/3	8	normal activity	GC(NCSE) 66.14 ± 9.71 vs 60.69 ± 11.33 vs 59.97 ± 8.46 Ex (TMT-B) -155.71 ± 98.17 VS -213.69 ± 93.16 VS -225.43 ± 80.24
Qi M 2019 (88)	MCI	26 vs16	70.6 ± 6.2 vs 69.1 ± 8.1	11/5 vs 12/4	Aerobic dance (MBE)	60-80%HRmax	35	3	13	usual care	GC(MMSE) 28.2 ± 1.0 vs 27.3 ± 1.7 Ex (TMT) -161.6 ± 53.8 vs -181.6 ± 46.7
Song D 2019 (89)	MCI	60vs60	76.22 ± 5.76 vs 75.33 ± 6.78	48/12 vs 42/18	Aerobic stepping exercise	Moderate intensity	60	3	16	health education	GC(MoCA) 23.66 ± 1.92 VS 21.40 ± 2.27
Tao, J. 2019 (90)	MCI	20 vs 17 vs 20	66.17 ± 4.17vs 64.32 ± 2.60vs 65.97 ± 5.66	15/5 vs 10/7 vs 14/6	(1) Baduanjin (MBE) (2) brisk walking (AE)	(1) NA (2) 55-75%HRR	60	3	24	health education	GC(MoCA) 2.10 ± 2.25 vs 2.88 ± 1.96 vs 1.10 ± 1.48
Tarumi, T. 2019 (91)	MCI	30 vs 39	64.0 ± 5.9 VS 65.3 ± 6.6	19/11 VS 24/15	AET: aerobic exercise treadmile	75–85% HRmax, (2 high intensity)	25-40	4.5	52	stretching and toning	Ex (D-KEFS -TMT) -11.9 ± 2.11 vs -12.4 ± 2.13
Bae, S.2019 (92)	MCI	41 vs 42	75.5 ± 6.0 VS 76.4 ± 5.1	18/23 VS 22/20	KENKOJISEICHI (physical, cognitive, and social activities) (ME)	Low intensity	75	<1	24	health education classes	GC(MMSE). 27.59 ± 2.68 vs 27.05 ± 2.55 Ex (TMT-A) 24.73 ± 9.11 vs 23.33 ± 7.22 Ex (TMT-B) 66.35 ± 47.23 vs 53.08 ± 37.59
Wang, L., et al., 2020 (93)	MCI	57 vs 54	68.37 ± 5.27 VS 68.24 ± 5.15	36/21 VS 32/22	Structured exercise: limbering-up, upper and lower limbs (ME)	60-80%HRmax	60	3	24	health promotion classes	GC(MoCA) 21.72 ± 2.19 vs 20.13 ± 2.43
Li, L.2021 (94)	MCI	42 vs 42	60 to 80 and above years	27/15 VS 24/18	Multi-component exercise: aerobic; strength; balance; coordination	Low intensity	30	5	25	community health instruction	GC(MMSE). 27.79 ± 1.18 vs 25.42 ± 2.28 (MoCA) 25.19 ± 1.29 vs 19.45 ± 2.00
Khanthong, P., et al., 2021 (95)	MCI	35 vs 36	60.36 ± 5.67 VS 61.47 ± 7.49	31/4 VS 25/11	RSD: 15 postures with 10 repetitions for each posture (MBE)		60	3	12	not RSD	GC(MoCA). 22.09 ± 3.47 vs 19.33 ± 2.77 Ex (TMT-A) -58.20 ± 21.27 vs -62.95 ± 25.78 Ex (TMT-B) -133.11± 69.60 vs -172.61± 83.88
Yu, A.P.2022 (96)	MCI	10 vs 12 vs 12	67.3 ± 4.2 VS 67.2 ± 6.8 VS 67.6 ± 8.1	7/3 VS 8/4 VS 10/2	(1) Taichi: standing; Yang-style Tai Chi; relaxation (MBE) (2) strength; aerobic; cooldown (ME)	(1) 3.24 METs (2) 4.3–5.5 METs	60	3	24	no intervention	GC(MoCA-HK) 25.0 ± 2.5 vs 26.6 ± 1.9 vs 18.9 ± 5.2 Ex (TMT-A) -11.9 ± 3.4 VS -10.7 ± 3.8 VS -16.0 ± 9.8
Li, F.2023 (97)	MCI	105 vs 107 vs 106	76.0 ± 5.1 VS 75.9 ± 5.1 VS 76.0 ± 6.1	75/30 VS 66/41 VS 71/35	(1) Cognitively Enhanced Tai Ji Quan (ME) (2) Tai Ji Quan (MBE)	Low intensity	60	2	24	Stretching Exercise	GC(MoCA) 28.3 ± 2.84 vs 27.0 ± 3.13 vs 25.3 ± 2.60 (TMT-B) -72.4 ± 2.0 VS -86.2 ± 2.0 VS -94.2 ± 2.1
Parial, L.L.2023 (98)	MCI	30 vs 30	63.33 ± 4.54 VS 64.27 ± 5.91	24/6 VS 22/8	Dual-task Zumba Gold: attention/orientation training; Zumba Gold dancing (ME)	Low intensity	60	3	12	moderate physical/leisure activities	GC(MoCA) 22.90 ± 2.30 vs 20.47 ± 2.6838 Ex (TMT-B) 204.35 ± 56.744 VS 199.98 ± 62.769
Uysal, İ.2023 (99)	MCI	12 vs 12	73.25 ± 2.01 VS 73.5 ± 3.21	2/10 VS 2/10	ADG: aerobic, dual-task and lower extremity strengthening exercises (ME)		30 + 30+NA	3	12	AG: no dual-task exercises (AE)	GC(MMSE) 23.17 ± 1.11 vs 22.75 ± 1.06
Zhang, Q.2023 (100)	MCI	14 vs 14 vs 14	66.67 ± 6.04 VS 66.22 ± 5.51 VS 69.75 ± 7.02	12/2 VS 13/1 VS 13/1	(1) TCE + RTG: stretching, aerobic (MBE) (2) Walking group (AE)	60% HRmax moderate	60	3	12	health knowledge	GC(MoCA) 23.28 ± 3.69 vs 20.89 ± 5.35 vs 19.59 ± 4.43 GC(MMSE) 25.78 ± 1.89 vs 23.56 ± 5.36 vs 22.71 ± 3.23

NA, not available; Dur, Duration; Freq, Frequence; Int, Intensity; Len, length; BI, Barthel Index; MMSE, Mini-Mental State Examination; MoCA, The Montreal cognitive assessment; Alzheimer’s Disease Assessment Scale-Cognitive Subscale (ADAS-Cog); TMT, Trial mark test; SCWT, Stroop color word test; FAB, Frontal Assessment Battery; the Wechsler Adult WAIS-III, Intelligence Scale 3rd Edition; ROCFT, Rey Osterrieth Complex Figure Test; EXIT-25, Executive Interview; ADCS-ADL, The Alzheimer’s Disease Cooperative Study Activities of Daily Living Inventory; FUCAS, Functional and Cognitive Assessment Test; KENKOJISEICHI:28 physical activities, 29 cognitive activities, and 44 social activities; NCSE, Neurobehavioral Cognitive Status Examination; SDMT, Symbol Digit Modalities Test; D-KEFS-TMT, Delis-Kaplan Executive function system trail making test score.

For all the outcomes, higher score means better function.

In the course of data extraction, we consulted the Chinese guidelines for the diagnosis and treatment of mild cognitive impairment to categorize cognitive function, and referenced the Physical Activity Guidelines for Americans and prior systematic reviews for the classification of exercise interventions (40, 41). To assess the impacts of different types of exercise interventions, we categorized exercise interventions into four hierarchical levels. First, interventions were classified as either “Exercise” or “Control” at the initial level. At the subsequent class level, interventions were further categorized based on their primary exercise type: (1) Aerobic Exercise (AE) aimed at enhancing cardiovascular fitness through activities like walking, running, or cycling; (2) Resistance Exercise (RE) focused on increasing muscular strength and power using equipment; (3) Multi-component Exercise (ME) Include more than two types of exercise, such as aerobic exercise, resistance exercise and other forms of training; and (4) Mind-Body Exercise (MBE) (aims to improve participants’ sense of mind-body coordination by emphasizing the interaction between the brain, body, mind, and behavior, such as Tai Chi); since the study control group we included was not all non-exercise group, but also included some slight movements and stretching, physical activity at different levels results in different improvements, and our study subjects are older people with mild cognitive impairment, since this group is sensitive to slight exercise, so the control were coded: (5) Passive control (the passive control group only studied courses such as health education); (6) Active control (the active control group did some daily activities or stretching exercises). The purpose of explore the optimal exercise types of the effects (**Appendix 2**).

Exercise duration in minutes was calculated for each study by considering factors such as program duration (in weeks), session duration (in minutes), frequency, intensity, week total, and overall total (in minutes). For instance, if intensity was described using the rate of perceived exertion (RPE) Borg scale (42), the corresponding heart rate was determined in alignment with the guidelines set forth by the American College of Sports Medicine (43).

### 2.4 Risk of bias assessment

The risk of bias in included randomized controlled trials was evaluated independently by two reviewers (YY and WJ) using the Cochrane risk of bias tool, which considers random sequence generation, allocation concealment, blinding of participants and personnel, blinding of outcome assessment, incomplete outcome data, selective reporting, and other biases. Disagreements were resolved through discussion and consultation with a third author (XJ), with each item being categorized as low, high, or unclear risk of bias (44).

### 2.5 Data synthesis and analysis

Prior to conducting the NMA, we evaluated three hypotheses as delineated in reference (45). The first hypothesis pertains to similarity, positing that baseline study characteristics should be comparable across studies incorporated into the NMA. To assess similarity, we examined whether the study design and the baseline conditions of the study participants were analogous. All studies included in our analysis were randomized controlled trials involving older adults diagnosed with mild cognitive impairment. The second factor is heterogeneity, which assumes the absence of variability in the results of pairwise comparisons. The third factor pertains to inconsistencies, implying no significant discrepancies between direct and indirect evidence (46).

We firstly performed a pairwise meta-analysis to examine how different exercise interventions compared to the control group across all outcomes. On the basis of postintervention scores, standardized mean differences (SMDs) and 95% confidence intervals (95%CIs) were calculated using a random effects model. If standard deviations (SDs) were not available, they were calculated from standard errors (SEs), CIs or p values, or the authors were contacted for missing data (47). The GetData Digitizer version 2.20 software was used to extract data from graphs in cases where the author did not report the data in the paper but provided a graph with the data. Finally, this review will use Review Manager 5.3 software and Stata 15.1 software for data analysis.

In Stata 15.1 software, frequentist NMA were conducted for the outcomes (48, 49). Through NMA, a network diagram is created, with each node representing one intervention and connecting lines between them representing one or more RCTs directly comparing the two interventions (50). The size of each node in the network diagram is proportionally weighted based on the number of participants receiving a specific intervention, while the thickness of the connecting lines between nodes is weighted according to the number of studies directly comparing the interventions (51). A random effects model is employed to address heterogeneity from other sources and produce more conservative confidence intervals for pooled effect estimates (52). The model is employed to address heterogeneity stemming from variations in cognitive and executive function measurement tools and other sources, yielding a more cautious confidence interval for combined point estimates. SMDs and 95% CIs were derived from post-intervention endpoint data to gauge the magnitude of the continuous outcome (53). SMDs and corresponding 95% CIs were calculated using post-intervention endpoint data to estimate the effect size of continuous outcomes. Cochrane classified effect sizes as small (SMD<0.40), medium (SMDs = 0.40-0.70), and large (SMDs > 0.70) (54). The inclusion of network transitivity as a crucial assumption in our analysis is deemed essential, as its evaluation directly influences the study outcomes (55). To ensure comparability among multiple treatment comparisons, we conducted a thorough examination of clinical and methodological characteristics, encompassing patient demographics and experimental designs, across all included studies (56). All of our studies were RCTs involving older adults with mild cognitive impairment. It was determined whether exercise interventions in the network were ranked by using the surface under the Cumulative Ranking Curve (SUCRA) as well as the average ranking. The higher the SUCRA value, the higher the ranking (57). The I² value was used to assess inter-study heterogeneity. Inconsistencies in global design and local design are detected using the design by process model and the loop-specific method (58). An analysis of publication bias was conducted using funnel plots.

We divided the articles involving exercise dose into two subgroups according to the two outcome indicators of overall cognition and executive function, extracted the data of the dose parameters in the articles. By analyzing subgroups, we extract the duration per session, frequency, length of intervention, intensity, weekly total time, total time and examine the proportional response to a given dose.

## 3 Results

### 3.1 Study selection

**Figure 1:**
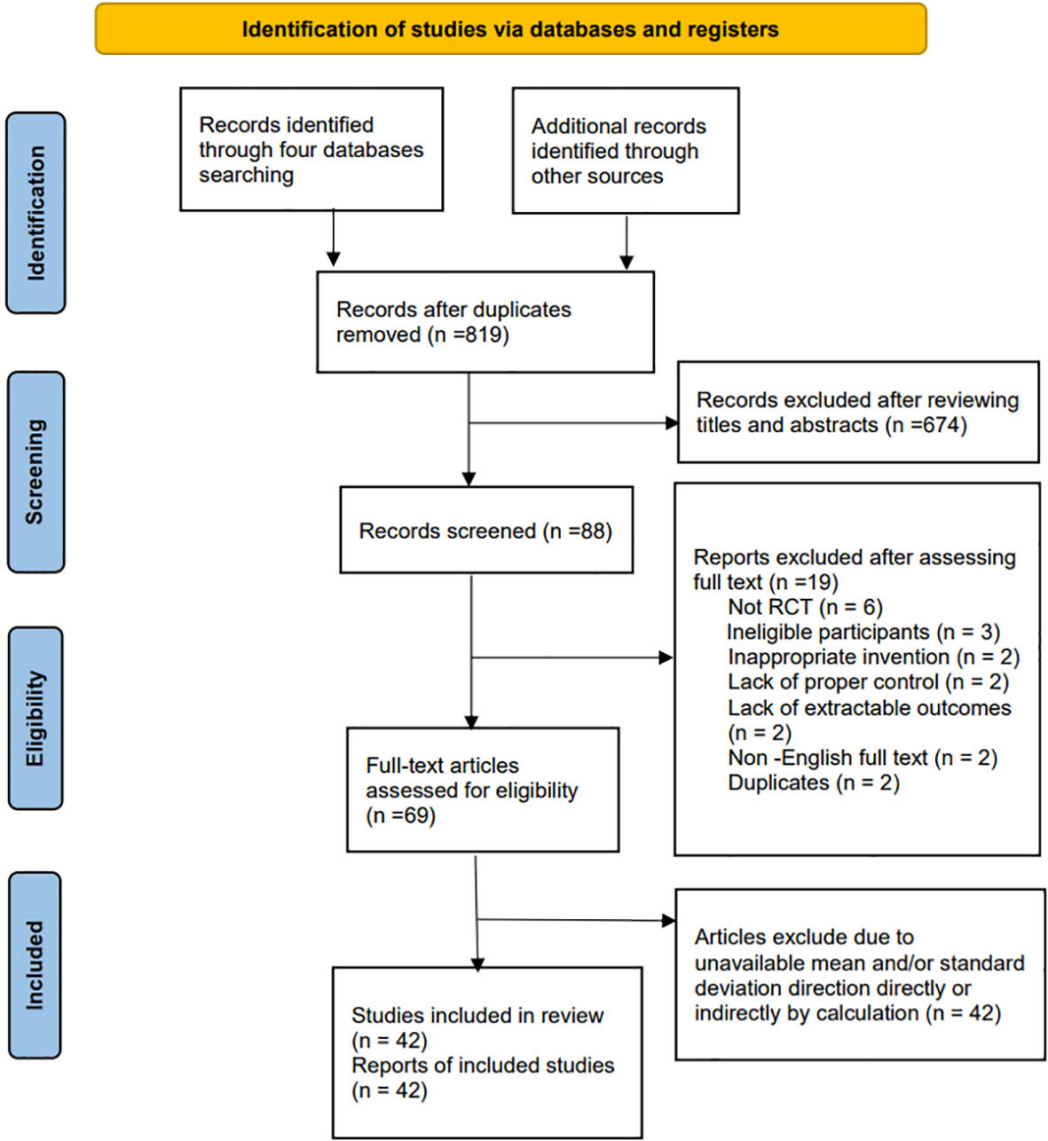
Flow diagram.

As a result of the search strategy, 847 articles initially appeared, of which 819 remained once duplicates were removed. After a thorough examination of titles and abstracts, 28 articles were excluded. Subsequently, 42 articles meeting the inclusion and exclusion criteria, published between 2003 and 2024 and involving 2832 participants, were chosen for network meta-analysis. The search and study selection process is illustrated in **Figure 1**.

### 3.2 Study characteristics

**Figure 2:**
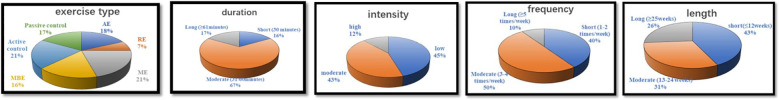
Percentage of intervention and exercise dose. AE, aerobic exercise; RE, resistant exercise; MBE, mind-body exercise; and ME, multi-component exercise.

**Table 1** displays the characteristics of the 42 randomized controlled trials conducted between 2004 and 2024. These trials included 13 studies (270 participants) investigating the effects of aerobic exercise, 5 studies (105 participants) examining resistance exercise effects, 15 studies (370 participants) studying multi-component exercise effects, 12 studies (447 participants) analyzing mind-body exercise effects, 14 studies (575 participants) exploring active control effects, and 24 studies (684 participants) investigating passive control effects. Furthermore, we included charts in **Table 1** to illustrate the varying exercise interventions and doses utilized in the studies, highlighting the percentage of each entry in relation to the total. This visual representation effectively demonstrates the divergent dose responses observed across the included interventions (**Figure 2**).

### 3.3 Dose-response descriptions in the included articles

We also perform the descriptive statistics for the dose-parameters of all MCI populations with optimal types of exercises, which was operationalized by considering various factors such as frequency (number of sessions per week), duration per session, length of intervention, exercise intensity (percentage of time spent exercising in heart rate), weekly total (time spent exercising per week), and overall total (total time spent exercising) (45). The findings of the study on the impact of multi-component exercise interventions on individuals with mild cognitive impairment indicated that programs characterized by short duration per session (30min), moderate frequency (3-4 times/week), moderate length of intervention (12-24 weeks), moderate intensity (60-85%HRmax etc.), total of ≥150 minutes per week, and total of >2160 minutes in overall total significantly correlated with higher effect sizes in enhancing the global cognition of the MCI population. Additionally, interventions with moderate duration per session (31-60min), moderate frequency, long length of intervention (≥25weeks), moderate intensity, total of ≥150 minutes per week, and total of >2160 minutes in overall total were found to be associated with higher effect sizes in improving the executive function of MCI patients.

### 3.4 Outcome measures

There were 34 studies measuring global function with a Mini-Mental State Examination (MMSE) (60–62, 65, 67, 69, 70, 73, 74, 80–82, 84, 86, 88, 92, 94, 99, 100), The Montreal cognitive assessment (MoCA) (68, 75, 77, 79, 83, 89, 90, 93, 95–98), Alzheimer’s Disease Assessment Scale-Cognitive Subscale (ADAS-Cog) (64, 66), Neurobehavioral Cognitive Status Examination (NCSE) (87).

Meanwhile, there were 15 studies using Trial mark test (TMT) for executive function assessment, which includes TMT-A, TMT-B and TMT-A+B (59, 61, 66, 74, 78–80, 87, 88, 92, 95–98). And 1 study used Stroop color word test (SCWT) (62), 1 study used Frontal Assessment Battery (FAB) (72), 1 study used Intelligence Scale 3rd Edition (WAIS-III) (64), 4 studies used Stroop test (63, 76, 77, 84), 2 studies used composite/Visuospatial test (68, 71), 1 study used Rey Osterrieth Complex Figure Test (ROCFT) (70) and 1 study used D-KEFS-TMT (91), which 26 studies totally with regard to executive function.

### 3.5 Risk of bias

The distribution of studies with varying levels of bias risk for specific components, including random sequence generation, allocation concealment, blinding of outcome assessors, incomplete outcome reporting, selective outcome reporting, and other risks of bias, is outlined as follows: random sequence generation (66.7%, 33.3%, and 0%, respectively); allocation concealment (52.4%, 45.2%, and 2.4%, respectively); blinding of outcome assessors (59.5%, 38.1%, and 2.4%, respectively); incomplete outcome (73.8%, 19.1%, and 7.1%, respectively); selective outcome reporting (81%, 9.5%, and 9.5%, respectively); and other risks of bias (61.9%, 33.3%, and 4.8%, respectively). Additional details regarding the bias risks of the included studies can be found in the **Appendix 3**.

### 3.6 Effect on global function improvement

#### 3.6.1 Optimal type of exercise

**Figure 3:**
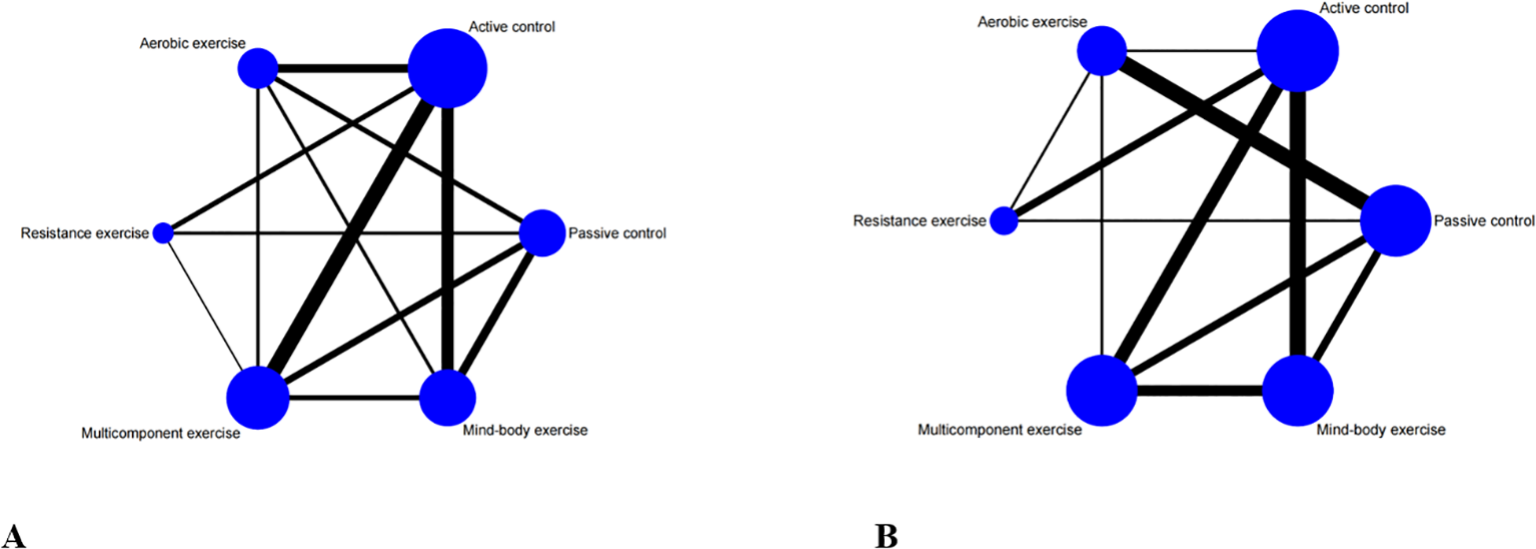
Network meta-analysis of eligible comparisons for **(A)** global cognition, **(B)** executive function. Each node represents an intervention, and the connecting lines between 2 nodes represents 1 or more randomized clinical trials (RCTs) in which the 2 interventions have been compared directly. The size of each node is proportional to the number of randomly assigned participants, and the thickness of the lines connecting 2 nodes is weighted according to the number of RCTs that directly compared the interventions it connected.

**Figure 4:**
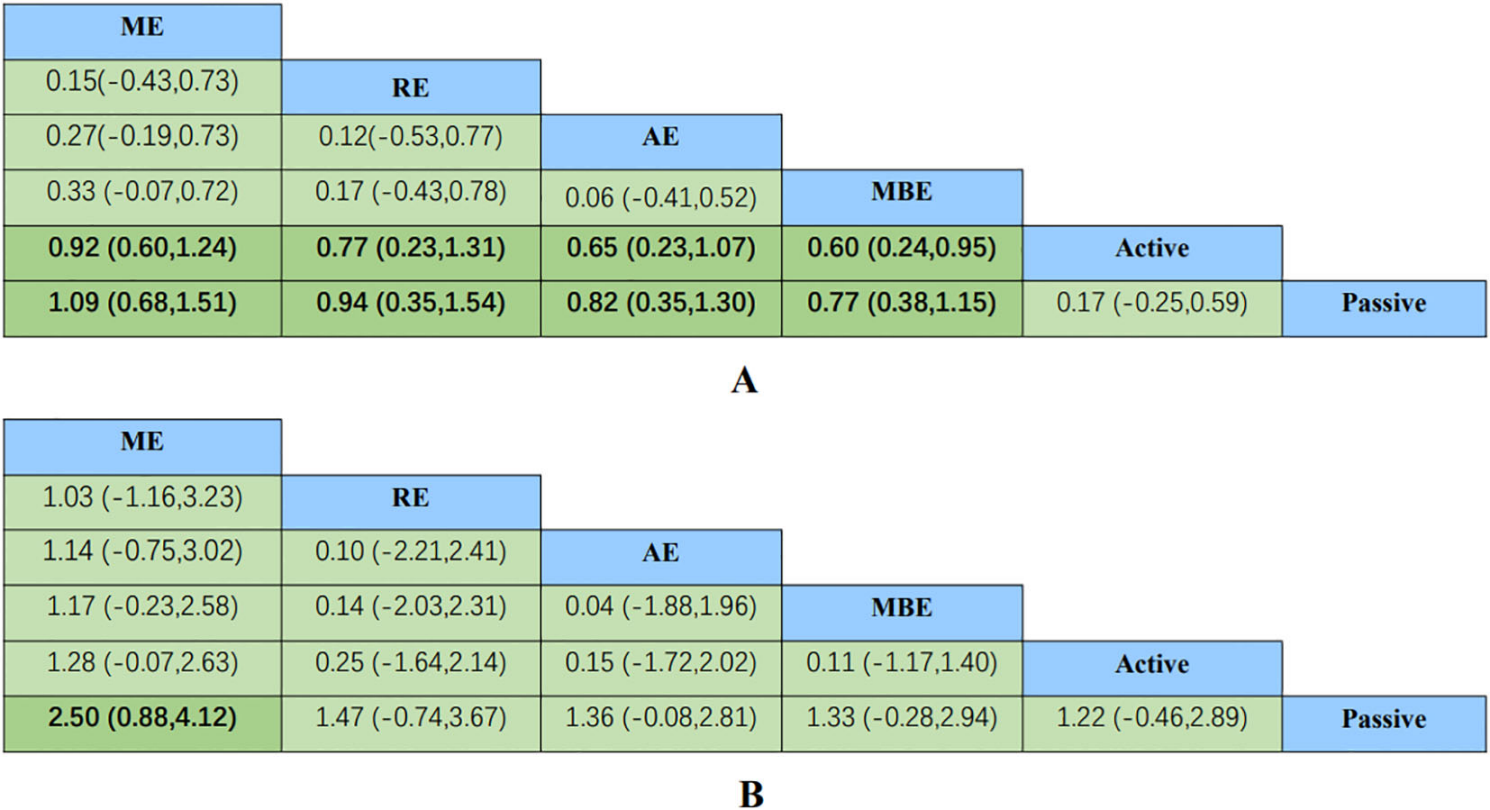
Comparative effectiveness results for **(A)** global cognition, **(B)** executive function. Each cell shows an SMD with a 95%CI. For any cell, a positive SMD favors the upper-left intervention; a negative SMD favors the lower-right intervention. 95%CI, 95% confidence interval; AE, aerobic exercise; MBE, mind body exercise; ME, multicomponent exercise; RE, resistance exercise; SMD, standardized mean difference.

**Figure 5:**
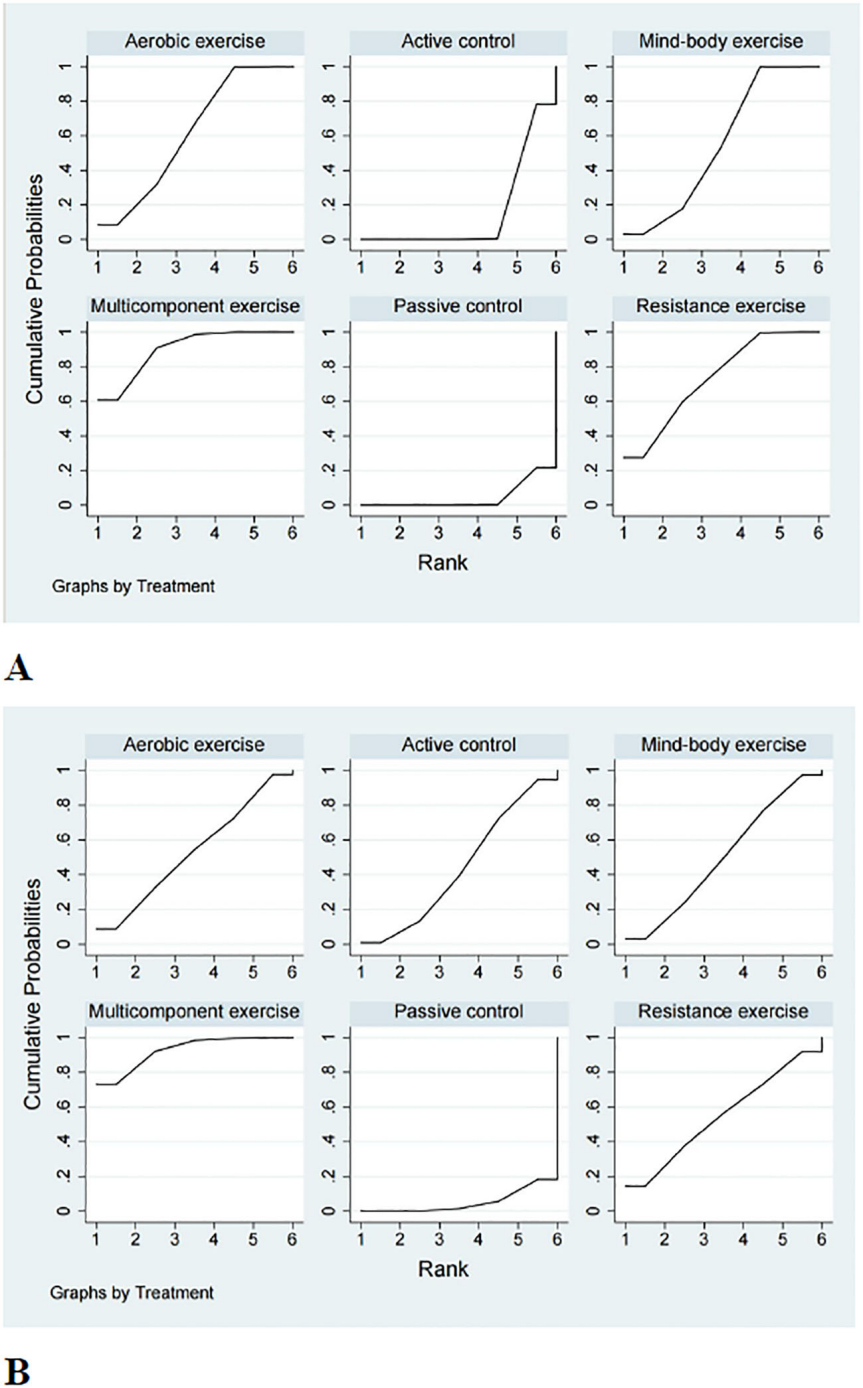
Cumulative ranking probability plots for **(A)** global cognition and **(B)** executive function. The horizontal axis represents the possible rank of each treatment (from best to worst according to the outcome). The vertical axis represents the cumulative probability for each treatment to be the best option, the best of 2 options, the best of 3 options, and so on.

A comprehensive analysis of global cognitive function was conducted, encompassing 34 studies involving a total of 2434 participants. Specifically, 8 studies investigated the impact of aerobic exercise, 5 studies assessed the effects of resistance exercise, 13 studies evaluated the influence of multi-component exercises, and 12 studies explored the effects of mind-body interventions. Meanwhile, we also classified the control group into active control and passive controls. Pairwise analysis demonstrated the effectiveness of exercise interventions compared to controls, while a network analysis inconsistency test did not reveal any significant global inconsistencies. Detailed results for inconsistency can be found in the **Appendix 4**. For comparisons between exercise interventions and controls on global cognition, including AE, RE, ME, and MBE, traditional meta-analysis forest plots were used. In **Figure 3**, all exercise interventions in the study were directly compared to control groups in the network plot for global cognition. The network meta-analysis results showed that exercise interventions, including RE, AE, and MBE, were more effective than both active and passive controls. The standardized mean differences ranged from 0.77 to 1.09 for comparisons with passive controls and from 0.60 to 0.92 for comparisons with active controls. The comparative impacts of various exercise interventions are depicted in **Figure 4**, with **Figure 5** displaying the ranking of exercise interventions based on cumulative probability plots and SUCRAs. The exercise intervention of multi-component exercise demonstrated the highest likelihood (60.7%) of being the most effective exercise type for enhancing global cognition in individuals with mild cognitive impairment, with a SUCRA value of 90.1% (**Table 2**). Analysis of the funnel plot did not reveal any significant publication bias and the heterogeneity of loops can be tested using loop-specific tests (**Appendix 4** **).**

**Table 2:** The global cognition and executive function rankings for different types of exercise.

Exercise	Global cognition			Executive function		
	SUCRA (%)	Mean rank	P (%)	SUCRA (%)	Mean rank	P (%)
AE	61.6	2.9	8.6	53.2	3.3	8.7
RE	73.3	2.3	27.5	54.8	3.3	14.5
ME	90.1	1.5	60.7	92.6	1.4	72.9
MBE	54.9	3.3	3.1	50.2	3.5	3.0
Active control	15.7	5.2	0.0	44.1	3.8	0.9
Passive control	4.4	5.8	0.0	5.2	5.7	0.0

Higher SUCRA and lower mean ranks indicate better-performing treatments. P indicates the probability of it being the best treatment. AE, aerobic exercise; MBE, mind body exercise; ME, multi-component exercise; RE, resistance exercise; SUCRA, surface under cumulative ranking curve.

#### 3.6.2 Dose response analysis of the effect of multi-component exercise on global functional

An analysis of dose response was conducted based on the duration per session, frequency, length of intervention, intensity, weekly total time, and total time of multi-component exercise per week.

The results showed that the improvement degree of multi-component exercise on global cognitive function was higher than that of control group, and the difference was statistically significant (P < 0.05). Exercise duration per session 30min group, duration per session 30-61min group, frequency 1-2 times/week group, frequency 3-4 times/week group, frequency≥5 times/week group, length of intervention ≤ 13weeks group, length of intervention 14-24 weeks group, length of intervention≥25 weeks group, low intensity group, moderate intensity group, week total<150min, week total ≥150min group, overall total ≤ 2160min group, overall total>2160min group, which scores after multi-component exercise intervention were superior to control groups, and the statistically significant difference were found (P < 0.05). Duration per session≥61min group was no statistical difference between exercise intervention and control group (P>0.05) (**Table 3**).

**Table 3:** pooled effect size test of the global cognition on multi-component exercise in MCI.

subgroup	Study number	Study research	Test of overall effect		
			SMD (95%CI)	Z	P
Duration per session					
Short (30 minutes)	2	144	1.04 (0.48,1.59)	3.68	<0.001
Moderate (31-60minutes)	8	559	0.90 (0.66,1.14)	7.35	<0.001
Long (≥61minutes)	3	193	0.15 (-0.19,0.49)	0.86	0.391
Frequency					
Short (1-2 times/week)	7	497	0.59 (0.22,0.97)	3.14	0.002
Moderate (3-4 times/week)	4	255	1.56 (0.62,2.49)	3.26	0.001
Long (≥5 times/week)	2	144	1.04 (0.48,1.59)	3.68	<0.001
Length of intervention					
short (≤13weeks)	4	159	0.73 (0.40,1.05)	4.39	<0.001
Moderate (14-24 weeks)	6	543	1.22 (0.64,1.81)	4.08	<0.001
Long (≥25weeks)	3	194	0.71 (0.002,1.42)	1.97	0.049
Intensity					
low	8	630	0.74 (0.42,1.05)	4.54	<0.001
moderate	5	266	1.32 (0.47,2.16)	3.05	0.002
Week total					
<150min	6	447	0.69 (0.33,1.06)	3.69	<0.001
≥150min	7	449	1.16 (0.60,1.73)	4.04	<0.001
Total					
≤2160min	5	242	0.55 (0.23,0.88)	3.34	0.001
>2160min	8	654	1.15 (0.70,1.60)	5.04	<0.001

### 3.7 Effect on executive function improvement

#### 3.7.1 Optimal type of exercise

A total of 26 studies focusing on executive function were included in the analysis, encompassing a total of 2003 participants. Among these studies, 7 investigated the impact of aerobic exercise, 4 examined resistance exercise, 8 explored multi-component exercise, and 9 delved into mind-body exercise, with controls categorized as active and passive controls in a consistent manner. Pairwise analysis demonstrated the efficacy of exercise interventions in enhancing executive function. A traditional meta-analysis was conducted, including forest plots of AE, RE, ME, and MBE interventions for comparison with each other and with control groups. Detailed results are provided in the (**Appendix 4**). According to **Figure 3**, direct comparisons between exercise interventions and controls are shown, but none are shown between RE and ME, RE and MBE, or AE and MBE. According to the network meta-analysis, exercise interventions demonstrated superior efficacy over control groups, with standardized mean differences ranging from 1.33 (95% confidence interval [CI]: -0.28 to 2.94) for multi-component exercise to 2.50 (95% CI: 0.88 to 4.12) for mind-body exercise when compared to passive controls. The comparative effectiveness of various exercise interventions is demonstrated in **Figure 4**, with the ranking of these interventions based on cumulative probability plots and Surface Under the Cumulative Ranking values shown in **Figure 5** and a corresponding **Table 2**. The exercise modality of multi-component exercise displayed the highest probability (72.9%) of being the most effective in enhancing executive function among individuals with mild cognitive impairment, with a SUCRA value of 92.6%. Additionally, no significant publication bias was observed based on the funnel plot (**Appendix 4**).

#### 3.7.2 Dose response analysis of the effect of multi-component exercise on executive functional

Dose response analysis was conducted according to the duration per session, frequency, length of intervention, intensity, weekly total time and total time of multi-component exercise.

According to the results, multi-component exercise improved executive function more than the control group, and the difference was statistically significant (P<0.05). Exercise frequency 1-2 times/week group, frequency 3-4 times/week group, length of intervention 14-24 weeks group, low intensity group, moderate intensity group, week total<150min, week total ≥150min group, total ≤ 2160min group, total>2160min group, which scores after multi-component exercise intervention were superior to those of control group, and the statistically significant difference were found (P < 0.05). Duration per session 31-60min group, duration per session≥61min group, length of intervention ≤ 13weeks group, length of intervention≥25 weeks group, were no statistical difference between exercise intervention and control group (P>0.05) (**Table 4**).

**Table 4:** pooled effect size test of the executive function on multi-component exercise in MCI.

subgroup	Study number	Study research	Test of overall effect		
			SMD (95%CI)	Z	P
Duration per session					
Short (30 minutes)	0				
Moderate (31-60minutes)	5	346	2.45 (-0.73,5.62)	1.51	0.131
Long (≥61minutes)	3	149	0.55 (-0.14,1.24)	1.56	0.119
Frequency					
Short (1-2 times/week)	5	395	0.30 (0.05,0.60)	1.99	0.046
Moderate (3-4 times/week)	3	100	1.21 (0.08,2.33)	2.11	0.035
Long (≥5 times/week)	0				
Length of intervention					
short (≤13weeks)	3	109	0.29 (-0.11,0.69)	1.41	0.159
Moderate (14-24 weeks)	3	320	0.40 (0.01,0.78)	2.03	0.042
Long (≥25weeks)	2	66	0.91 (-0.79,2.62)	1.05	0.293
Intensity					
low	5	436	0.28 (0.01,0.54)	2.04	0.041
moderate	3	59	1.21 (0.08,2.33)	2.11	0.035
Week total					
<150min	4	345	0.37 (0.02,0.72)	2.07	0.038
≥150min	4	150	1.21 (0.08,2.33)	2.11	0.035
Total					
≤2160min	4	192	0.30 (0.01,0.58)	2.01	0.044
>2160min	4	303	1.87 (0.67,3.08)	3.04	0.002

## 4 Discussion

Exercise interventions for improving global cognition and executive function in mild cognitive impairment patients were investigated in this network meta-analysis (n=2832, 42 studies) (101). Our findings suggest multi-component exercise and resistance exercise as the most beneficial interventions for both outcomes. Multi-component exercise combines at least two modalities, such as aerobic and resistance training (102). While ME offers potential for broader neurobiological benefits (e.g., BDNF, IGF-1), its efficacy might be hindered by logistical challenges. Balancing optimal duration and frequency for each component within a complex intervention can be difficult, potentially diminishing the overall effect. Additionally, implementing ME requires careful consideration of its multifaceted nature. Despite these challenges, ME emerged as the most effective intervention for enhancing executive function in MCI. This is likely due to its ability to directly target executive skills through diverse motor tasks involving sensorimotor adaptation and neuromuscular coordination (103). However, the optimal combination, frequency, and duration of individual components within ME remain unclear and warrant further investigation. Further research is needed to define the ideal ME structure and expand upon this promising approach (104). Notably, direct comparisons between interventions provide more reliable evidence than indirect comparisons, highlighting the need for future multi-group studies (105).

In light of the analysis techniques employed in published literature on exercise dosage and the quantity of articles addressing this topic, a subgroup analysis was conducted on the effects of multi-component exercise dosage on the cognitive and executive function of individuals with mild cognitive impairment. We studied published articles and conducted subgroup analyses of outcome indicators. Since our outcome indicators were overall cognition and executive function, we analyzed these subgroups separately. Based on the established parameters for time allocation in traditional exercise intervention studies, session duration is categorized into three groups: 30 minutes, 30-61 minutes, and≥61 minutes. Frequency of sessions is then determined based on the included literature, with groups ranging from 1-2 times per week, 3-4 times per week, and≥5 times per week (35). In randomized controlled trials, the minimum intervention period is 8 weeks, with optimal results achieved through longer intervention durations. The included literature is further categorized into intervention periods of ≤13 weeks, 14-24 weeks, and ≥25 weeks. World Health Organization (WHO) guidelines recommend that adults engage in 150 to 300 minutes of moderate-intensity physical activity each week, or 75 to 150 minutes of vigorous-intensity activity each week (106). The classification of exercise intensity is determined by factors such as maximum heart rate and the type of exercise performed. In consideration of the elderly individuals with mild cognitive impairment comprising the subjects of our study, we opted to utilize the specified minimum criteria to categorize participants into groups based on their total weekly exercise duration as either <150 minutes or ≥150 minutes. The World Health Organization recommends engaging in physical exercise at least three days per week for a duration of 60 minutes (107), based on the standard of at least 150 minutes of exercise per week. Most exercise interventions typically last between 8-12 weeks, with the latter duration chosen in accordance with the literature reviewed. As such, the classification index is calculated as 60 minutes per session, three sessions per week, and 12 weeks, resulting in a total of 2160 minutes. Stata15.1 software was utilized to compute the articles included in the aforementioned items, resulting in the determination of the SMDs, 95%CIs, Z value, and P value. The derived values were subsequently examined and evaluated to ascertain their significance, as well as to identify the optimal exercise dosage for each item.

Due to the limited number of studies available for classification (only two studies) and the heterogeneous nature of the literature (108), it is important to note that the definition of physical activity (at least three times a week for 30 minutes) closely aligns with the current recommendations set forth by the World Health Organization (150 minutes per week) (109). The study included in the group with sessions lasting 61 minutes or more, conducted only 1-2 times per week, did not meet the minimum standards outlined by the World Health Organization. As a result, the effectiveness of this group was not as significant as the groups with shorter session durations. The World Health Organization (WHO) classifies individuals aged 65 and older as elderly (110). Given that all participants in this study are individuals diagnosed with mild cognitive impairment who fall within this age range, it is important to consider that longer exercise sessions may lead to fatigue, ultimately diminishing the effectiveness of the exercise regimen (111). Therefore, optimizing the duration of each session is crucial for maximizing the benefits of exercise (112). Additionally, excessive frequency of exercise without adequate rest may result in fatigue and hinder the body’s ability to fully recover (113), ultimately diminishing the overall impact of the exercise routine (114, 115). For length of intervention, the longer the exercise, the better the results (116). The quantity of articles with intervention periods exceeding 25 weeks is limited, with only one study spanning 52 weeks, a duration significantly longer than the majority of articles. The remaining articles have intervention periods of 25 and 26 weeks, which are in close proximity to the standard 24-week duration. The exercise effects observed in these studies are reported to be largely similar, making direct comparisons challenging. Additionally, the study with a 52-week intervention period involved only two exercise sessions per week, contrasting with the more common 3-4 sessions per week in other studies. This discrepancy raises the possibility that prolonged exposure to light exercise may lead to adaptability and decreased motivation among participants, potentially impacting the overall efficacy of the exercise regimen (117).

The quantity of articles addressing executive function was deemed inadequate, potentially leading to heterogeneity (P > 0.05) and a lack of information on session duration. Similarly, in relation to global cognition, optimal exercise duration can effectively maintain physical well-being without imposing undue psychological strain on patients, yielding excellent results (118). In terms of intervention duration, post-reclassification revealed a limited number of articles (2-3) with uncontrolled heterogeneity, thereby hindering the elucidation of the most effective intervention length (119). Future studies should further expand the number of included articles and analyze them to obtain reliable research results (120, 121).

Our network meta-analysis is subject to several limitations. Firstly, inherent heterogeneity in sports intervention and potential changes in practice may impact the results (122). Secondly, the use of diverse assessment tools to measure cognitive function could further contribute to heterogeneity. Additionally, limitations in available data from previous trials prevented the evaluation of exercise effects on other cognitive domains, and the reliability of exercise dose extraction in overall cognitive function and executive function remains uncertain. Our study specifically examined an elderly population with mild cognitive impairment, however, the literature reviewed lacked detailed subtyping or clear indications of specific subtypes within this population. As a result, we were unable to perform more thorough subgroup analyses of our participants or target specific cognitive functions for improvement with greater accuracy. Finally, the availability of articles containing follow-up data is restricted, hindering our ability to conduct statistical analysis on such data.

Moving forward, our objective is to classify individuals with mild cognitive impairment into specific subtypes, including amnestic mild cognitive impairment (aMCI) as well as single-domain and multi-domain mild cognitive impairment (sdMCI and mdMCI) (123). These distinct subtypes demonstrate diverse reactions to various forms of exercise and exercise routines. Thus, it is crucial to accurately categorize patients with mild cognitive impairment to improve cognitive function and attain an optimal cognitive state. Given a sufficient number of articles, a subgroup analysis based on gender is conducted on the included research subjects. The varying physiological structures of women and men result in divergent effects from the same exercise intervention (124). Additionally, the limited availability of articles containing follow-up data poses a challenge to conducting statistical analysis on this information, thereby impeding the evaluation of sustained cognitive enhancement post-intervention. Consequently, our future research efforts will focus on investigating the influence of follow-up duration on cognitive improvement. Concurrently, our forthcoming research endeavors should prioritize the sustainability of long-term follow-up on cognitive function enhancement. The incorporated literature encompasses follow-up data, which will be scrutinized and deliberated upon to bolster the credibility of research findings. It is imperative to incorporate additional articles that provide comprehensive descriptions of exercise dosage in order to bolster the quantity of literature and the reliability of data, thereby aligning with the parameters of the restricted cubic spline plot and enhancing the validity of the research findings. Finally, it is imperative to prioritize the psychological well-being of research subjects, as the psychological dimension serves as a mediator that can significantly influence their physical health, warranting careful consideration (125).

## 5 Conclusion

This network meta-analysis has shown that multi-component exercise resulted in more positive outcomes in terms of global cognitive and executive function in patients with mild cognitive impairment. However, it is essential to approach these findings with caution due to the limitations of the meta-analysis methodology and the limited number of studies in the existing literature. The present review does not definitively establish the ideal exercise regimen for individuals with mild cognitive impairment. Consistent with prior recommendations, engaging in moderate intensity multi-component exercise sessions at least three times weekly, with shorter durations and increased frequencies of such exercises, is likely to yield the most favorable cognitive outcomes. Striking a proper balance is crucial, and integrating multi-component exercise into daily routines can lead to enduring benefits. Future research should focus on conducting additional randomized controlled trials to offer more conclusive evidence regarding the comparative effectiveness of various exercise interventions.

## Data Availability

The original contributions presented in the study are included in the article/**Supplementary Material**. Further inquiries can be directed to the corresponding author.

## References

[1] Shen J Feng B Fan L Jiao Y Li Y Liu H . Triglyceride glucose index predicts all-cause mortality in oldest-old patients with acute coronary syndrome and diabetes mellitus. BMC Geriatr. (2023) 23:78. doi: 10.1186/s12877-023-03788-3 36747129 PMC9901061

[2] Chojnacki C Gąsiorowska A Popławski T Konrad P Chojnacki M Fila M . Beneficial effect of increased tryptophan intake on its metabolism and mental state of the elderly. Nutrients. (2023) 15(4):847. doi: 10.3390/nu15040847 PMC9961537 36839204

[3] Levine DA Galecki AT Morgenstern LB Zahuranec DB Langa KM Kabeto MU . Preexisting mild cognitive impairment, dementia, and receipt of treatments for acute ischemic stroke. Stroke. (2021) 52:2134–42. doi: 10.1161/STROKEAHA.120.032258 PMC8154649 33902296

[4] Aggarwal NT Tripathi M Dodge HH Alladi S Anstey KJ . Trends in Alzheimer’s disease and dementia in the asian-pacific region. Int J Alzheimers Dis. (2012) 2012:171327. doi: 10.1155/2012/171327 23304631 PMC3523465

[5] Wu YT Beiser AS Breteler MMB Fratiglioni L Helmer C Hendrie HC . The changing prevalence and incidence of dementia over time - current evidence. Nat Rev Neurol. (2017) 13:327–39. doi: 10.1038/nrneurol.2017.63 28497805

[6] Jia L Du Y Chu L Zhang Z Li F Lyu D . Prevalence, risk factors, and management of dementia and mild cognitive impairment in adults aged 60 years or older in China: a cross-sectional study. Lancet Public Health. (2020) 5:e661–e71. doi: 10.1016/S2468-2667(20)30185-7 33271079

[7] Jia J Wei C Chen S Li F Tang Y Qin W . The cost of Alzheimer’s disease in China and re-estimation of costs worldwide. Alzheimers Dement. (2018) 14:483–91. doi: 10.1016/j.jalz.2017.12.006 29433981

[8] Anderson ND . State of the science on mild cognitive impairment (MCI). CNS Spectr. (2019) 24:78–87. doi: 10.1017/S1092852918001347 30651152

[9] Falvo I Fiordelli M Amati R Ibnidris A Albanese E Fadda M . Participants’ Comprehension of the informed consent in an epidemiological study on dementia prevalence: A qualitative study. Front Psychiatry. (2021) 12:656822. doi: 10.3389/fpsyt.2021.656822 33897504 PMC8058191

[10] Gu J Li D Li Z Guo Y Qian F Wang Y . The effect and mechanism of transcranial direct current stimulation on episodic memory in patients with mild cognitive impairment. Front Neurosci. (2022) 16:811403. doi: 10.3389/fnins.2022.811403 35250453 PMC8891804

[11] Yong L Liu L Ding T Yang G Su H Wang J . Evidence of effect of aerobic exercise on cognitive intervention in older adults with mild cognitive impairment. Front Psychiatry. (2021) 12:713671. doi: 10.3389/fpsyt.2021.713671 34354619 PMC8329556

[12] Ehtewish H Arredouani A El-Agnaf O . Diagnostic, prognostic, and mechanistic biomarkers of diabetes mellitus-associated cognitive decline. Int J Mol Sci. (2022) 23(11):6144. doi: 10.3390/ijms23116144 PMC9181591 35682821

[13] Chang CH Yeh CH Chang CC Lin YC . Interactive somatosensory games in rehabilitation training for older adults with mild cognitive impairment: usability study. JMIR Serious Games. (2022) 10:e38465. doi: 10.2196/38465 35834303 PMC9335175

[14] Miyazaki A Mori H . Frequent karaoke training improves frontal executive cognitive skills, tongue pressure, and respiratory function in elderly people: pilot study from a randomized controlled trial. Int J Environ Res Public Health. (2020) 17(4):1459. doi: 10.3390/ijerph17041459 PMC7068312 32102472

[15] Beishon LC Batterham AP Quinn TJ Nelson CP Panerai RB Robinson T . Addenbrooke’s Cognitive Examination III (ACE-III) and mini-ACE for the detection of dementia and mild cognitive impairment. Cochrane Database Syst Rev. (2019) 12:Cd013282. doi: 10.1002/14651858.CD013282.pub2 31846066 PMC6916534

[16] Farina N Llewellyn D Isaac MG Tabet N . Vitamin E for Alzheimer’s dementia and mild cognitive impairment. Cochrane Database Syst Rev. (2017) 1:Cd002854. doi: 10.1002/14651858.CD002854.pub4 28128435 PMC6464807

[17] Yun S Ryu S . The effects of cognitive-based interventions in older adults: A systematic review and meta-analysis. Iran J Public Health. (2022) 51:1–11. doi: 10.18502/ijph.v51i1.8286 35223620 PMC8837877

[18] Abd-Alrazaq A Abuelezz I AlSaad R Al-Jafar E Ahmed A Aziz S . Serious games for learning among older adults with cognitive impairment: systematic review and meta-analysis. J Med Internet Res. (2023) 25:e43607. doi: 10.2196/43607 37043277 PMC10134019

[19] Petersen RC Lopez O Armstrong MJ Getchius TSD Ganguli M Gloss D . Practice guideline update summary: Mild cognitive impairment: Report of the Guideline Development, Dissemination, and Implementation Subcommittee of the American Academy of Neurology. Neurology. (2018) 90:126–35. doi: 10.1212/WNL.0000000000004826 PMC5772157 29282327

[20] Wang MC Liao WC Lee KC Lu SH Lin YP . Validation of screening tools for predicting the risk of functional decline in hospitalized elderly patients. Int J Environ Res Public Health. (2022) 19(11):6685. doi: 10.3390/ijerph19116685 PMC9180656 35682269

[21] Martínez-Velilla N Casas-Herrero A Zambom-Ferraresi F Sáez de Asteasu ML Lucia A Galbete A . Effect of exercise intervention on functional decline in very elderly patients during acute hospitalization: A randomized clinical trial. JAMA Intern Med. (2019) 179:28–36. doi: 10.1001/jamainternmed.2018.4869 30419096 PMC6583412

[22] Warburton DE Nicol CW Bredin SS . Prescribing exercise as preventive therapy. Cmaj. (2006) 174:961–74. doi: 10.1503/cmaj.1040750 PMC1405860 16567757

[23] Angevaren M Aufdemkampe G Verhaar HJ Aleman A Vanhees L . Physical activity and enhanced fitness to improve cognitive function in older people without known cognitive impairment. Cochrane Database Syst Rev. (2008) 2:Cd005381. doi: 10.1002/14651858.CD005381.pub2. 18425918

[24] Northey JM Cherbuin N Pumpa KL Smee DJ Rattray B . Exercise interventions for cognitive function in adults older than 50: a systematic review with meta-analysis. Br J Sports Med. (2018) 52:154–60. doi: 10.1136/bjsports-2016-096587 28438770

[25] Jung M Tu Y Park J Jorgenson K Lang C Song W . Surface-based shared and distinct resting functional connectivity in attention-deficit hyperactivity disorder and autism spectrum disorder. Br J Psychiatry. (2019) 214:339–44. doi: 10.1192/bjp.2018.248 PMC6521835 31088591

[26] Zhao X Jin Y Li H Jia Y Wang Y . Sevoflurane impairs learning and memory of the developing brain through post-transcriptional inhibition of CCNA2 via microRNA-19-3p. Aging (Albany NY). (2018) 10:3794–805. doi: 10.18632/aging.v10i12 PMC6326694 30540563

[27] Egan B Zierath JR . Exercise metabolism and the molecular regulation of skeletal muscle adaptation. Cell Metab. (2013) 17:162–84. doi: 10.1016/j.cmet.2012.12.012 23395166

[28] Yang J Dong Y Yan S Yi L Qiu J . Which specific exercise models are most effective on global cognition in patients with cognitive impairment? A network meta-analysis. Int J Environ Res Public Health. (2023) 20(4):2790. doi: 10.3390/ijerph20042790 PMC9957167 36833483

[29] Wang KH Wu JR Zhang D Duan XJ Ni MW . Comparative efficacy of Chinese herbal injections for treating chronic heart failure: a network meta-analysis. BMC Complement Altern Med. (2018) 18:41. doi: 10.1186/s12906-018-2090-3 29386000 PMC5793420

[30] Palacios-Cartagena RP Parraca JA Mendoza-Muñoz M Pastor-Cisneros R Muñoz-Bermejo L Adsuar JC . Level of physical activity and its relationship to self-perceived physical fitness in Peruvian adolescents. Int J Environ Res Public Health. (2022) 19(3):1182. doi: 10.3390/ijerph19031182 PMC8834742 35162206

[31] Ballester-Ferrer JA Carbonell-Hernández L Pastor D Cervelló E . COVID-19 quarantine impact on wellbeing and cognitive functioning during a 10-week high-intensity functional training program in young university students. Front Behav Neurosci. (2022) 16:822199. doi: 10.3389/fnbeh.2022.822199 35464146 PMC9028760

[32] Savaris RF Fuhrich DG Duarte RV Franik S Ross J . Antibiotic therapy for pelvic inflammatory disease. Cochrane Database Syst Rev. (2017) 4:Cd010285. doi: 10.1002/14651858.CD010285.pub2 28436019 PMC6478260

[33] Wang F Han J He Q Geng Z Deng Z Qiao D . Applying (1)H NMR spectroscopy to detect changes in the urinary metabolite levels of chinese half-pipe snowboarders after different exercises. J Anal Methods Chem. (2015) 2015:315217. doi: 10.1155/2015/315217 26101694 PMC4458538

[34] Krekeler BN Rowe LM Connor NP . Dose in exercise-based dysphagia therapies: A scoping review. Dysphagia. (2021) 36:1–32. doi: 10.1007/s00455-020-10104-3 32140905 PMC7483259

[35] Sanders LMJ Hortobágyi T la Bastide-van Gemert S van der Zee EA van Heuvelen MJG . Dose-response relationship between exercise and cognitive function in older adults with and without cognitive impairment: A systematic review and meta-analysis. PloS One. (2019) 14:e0210036. doi: 10.1371/journal.pone.0210036 30629631 PMC6328108

[36] Huang J Zheng Y Gao D Hu M Yuan T . Effects of exercise on depression, anxiety, cognitive control, craving, physical fitness and quality of life in methamphetamine-dependent patients. Front Psychiatry. (2019) 10:999. doi: 10.3389/fpsyt.2019.00999 32047445 PMC6997340

[37] Nerz C Kramer-Gmeiner F Jansen CP Labudek S Klenk J Becker C . Group-based and individually delivered liFE: content evaluation and predictors of training response - A dose-response analysis. Clin Interv Aging. (2022) 17:637–52. doi: 10.2147/CIA.S359150 PMC9057901 35509348

[38] Chang J Zhu W Zhang J Yong L Yang M Wang J . The effect of chinese square dance exercise on cognitive function in older women with mild cognitive impairment: the mediating effect of mood status and quality of life. Front Psychiatry. (2021) 12:711079. doi: 10.3389/fpsyt.2021.711079 34305689 PMC8298898

[39] Page MJ McKenzie JE Bossuyt PM Boutron I Hoffmann TC Mulrow CD . The PRISMA 2020 statement: an updated guideline for reporting systematic reviews. Bmj. (2021) 372:n71. doi: 10.1136/bmj.n71 33782057 PMC8005924

[40] Zou L Loprinzi PD Yeung AS Zeng N Huang T . The beneficial effects of mind-body exercises for people with mild cognitive impairment: a systematic review with meta-analysis. Arch Phys Med Rehabil. (2019) 100:1556–73. doi: 10.1016/j.apmr.2019.03.009 30986409

[41] Wang S Yin H Wang X Jia Y Wang C Wang L . Efficacy of different types of exercises on global cognition in adults with mild cognitive impairment: a network meta-analysis. Aging Clin Exp Res. (2019) 31:1391–400. doi: 10.1007/s40520-019-01142-5 30739298

[42] Arney BE Glover R Fusco A Cortis C de Koning JJ van Erp T . Comparison of RPE (Rating of perceived exertion) scales for session RPE. Int J Sports Physiol Perform. (2019) 14:994–6. doi: 10.1123/ijspp.2018-0637 30569764

[43] Bento-Torres J Bento-Torres NVO Stillman CM Grove GA Jr. Huang H Uyar F . Associations between cardiorespiratory fitness, physical activity, intraindividual variability in behavior, and cingulate cortex in younger adults. J Sport Health Sci. (2019) 8:315–24. doi: 10.1016/j.jshs.2019.03.004 PMC6620364 31333884

[44] Higgins JP Altman DG Gøtzsche PC Jüni P Moher D Oxman AD . The Cochrane Collaboration’s tool for assessing risk of bias in randomised trials. Bmj. (2011) 343:d5928. doi: 10.1136/bmj.d5928 22008217 PMC3196245

[45] Flack KD Hays HM Moreland J Long DE . Exercise for weight loss: further evaluating energy compensation with exercise. Med Sci Sports Exerc. (2020) 52:2466–75. doi: 10.1249/MSS.0000000000002376 PMC7556238 33064415

[46] Liu C Wang Y Yu W Xiang J Ding G Liu W . Comparative effectiveness of noninvasive therapeutic interventions for myofascial pain syndrome: a network meta-analysis of randomized controlled trials. Int J Surg. (2024) 110:1099–112. doi: 10.1097/JS9.0000000000000860 PMC10871620 37939115

[47] Yang J Chen J Yang M Yu S Ying L Liu GJ . Acupuncture for hypertension. Cochrane Database Syst Rev. (2018) 11:Cd008821. doi: 10.1002/14651858.CD008821.pub2 30480757 PMC6516840

[48] Huang X Zhao X Li B Cai Y Zhang S Wan Q . Comparative efficacy of various exercise interventions on cognitive function in patients with mild cognitive impairment or dementia: A systematic review and network meta-analysis. J Sport Health Sci. (2022) 11:212–23. doi: 10.1016/j.jshs.2021.05.003 PMC9068743 34004389

[49] Shim S Yoon BH Shin IS Bae JM . Network meta-analysis: application and practice using Stata. Epidemiol Health. (2017) 39:e2017047. doi: 10.4178/epih.e2017047 29092392 PMC5733388

[50] Gibbison B López-López JA Higgins JP Miller T Angelini GD Lightman SL . Corticosteroids in septic shock: a systematic review and network meta-analysis. Crit Care. (2017) 21:78. doi: 10.1186/s13054-017-1659-4 28351429 PMC5371269

[51] Desborough M Estcourt LJ Chaimani A Doree C Hopewell S Trivella M . Alternative agents versus prophylactic platelet transfusion for preventing bleeding in patients with thrombocytopenia due to chronic bone marrow failure: a network meta-analysis and systematic review. Cochrane Database Syst Rev. (2016) 2016(1):CD012055. doi: 10.1002/14651858.CD012055. PMC4826602 27069420

[52] Nalls MA Duran R Lopez G Kurzawa-Akanbi M McKeith IG Chinnery PF . A multicenter study of glucocerebrosidase mutations in dementia with Lewy bodies. JAMA Neurol. (2013) 70:727–35. doi: 10.1001/jamaneurol.2013.1925 PMC3841974 23588557

[53] Nakagawa A Watanabe N Omori IM Barbui C Cipriani A McGuire H . Milnacipran versus other antidepressive agents for depression. Cochrane Database Syst Rev. (2009) 2009:Cd006529. doi: 10.1002/14651858 19588396 PMC4164845

[54] Li CQ Wang YC Shen SQ Zhang YL Zhao JQ Zou WB . Effects of exercise by type and duration on quality of life in patients with digestive system cancers: A systematic review and network meta-analysis. J Sport Health Sci. (2023) 12:491–500. doi: 10.1016/j.jshs.2022.12.008 36528289 PMC10362486

[55] Cumpston M Li T Page MJ Chandler J Welch VA Higgins JP . Updated guidance for trusted systematic reviews: a new edition of the Cochrane Handbook for Systematic Reviews of Interventions. Cochrane Database Syst Rev. (2019) 10:Ed000142. doi: 10.1002/14651858 31643080 PMC10284251

[56] Chaimani A Vasiliadis HS Pandis N Schmid CH Welton NJ Salanti G . Effects of study precision and risk of bias in networks of interventions: a network meta-epidemiological study. Int J Epidemiol. (2013) 42:1120–31. doi: 10.1093/ije/dyt074 23811232

[57] Huang IH Wu PC Lee YH Kang YN . Optimal treatment strategy of fremanezumab in migraine prevention: a systematic review with network meta-analysis of randomized clinical trials. Sci Rep. (2020) 10:18609. doi: 10.1038/s41598-020-75602-8 33122778 PMC7596067

[58] Shim SR Kim SJ Lee J Rücker G . Network meta-analysis: application and practice using R software. Epidemiol Health. (2019) 41:e2019013. doi: 10.4178/epih.e2019013 30999733 PMC6635665

[59] Scherder EJ Van Paasschen J Deijen JB van der Knokke S Orlebeke JF Burgers I . Physical activity and executive functions in the elderly with mild cognitive impairment. Aging Ment Health. (2005) 9:272–80. doi: 10.1080/13607860500089930 16019281

[60] Varela S Ayán C Cancela JM Martín V . Effects of two different intensities of aerobic exercise on elderly people with mild cognitive impairment: a randomized pilot study. Clin Rehabil. (2012) 26:442–50. doi: 10.1177/0269215511425835 22116953

[61] Lam LC Chau RC Wong BM Fung AW Tam CW Leung GT . A 1-year randomized controlled trial comparing mind body exercise (Tai Chi) with stretching and toning exercise on cognitive function in older Chinese adults at risk of cognitive decline. J Am Med Dir Assoc. (2012) 13:568.e15–20. doi: 10.1016/j.jamda.2012.03.008 22579072

[62] Suzuki T Shimada H Makizako H Doi T Yoshida D Tsutsumimoto K . Effects of multicomponent exercise on cognitive function in older adults with amnestic mild cognitive impairment: a randomized controlled trial. BMC Neurol. (2012) 12:128. doi: 10.1186/1471-2377-12-128 23113898 PMC3534485

[63] Davis JC Bryan S Marra CA Sharma D Chan A Beattie BL . An economic evaluation of resistance training and aerobic training versus balance and toning exercises in older adults with mild cognitive impairment. PloS One. (2013) 8:e63031. doi: 10.1371/journal.pone.0063031 23690976 PMC3653911

[64] Fiatarone Singh MA Gates N Saigal N Wilson GC Meiklejohn J Brodaty H . The Study of Mental and Resistance Training (SMART) study—resistance training and/or cognitive training in mild cognitive impairment: a randomized, double-blind, double-sham controlled trial. J Am Med Dir Assoc. (2014) 15:873–80. doi: 10.1016/j.jamda.2014.09.010 25444575

[65] Wei XH Ji LL . Effect of handball training on cognitive ability in elderly with mild cognitive impairment. Neurosci Lett. (2014) 566:98–101. doi: 10.1016/j.neulet.2014.02.035 24582900

[66] Lü J Sun M Liang L Feng Y Pan X Liu Y . Effects of momentum-based dumbbell training on cognitive function in older adults with mild cognitive impairment: a pilot randomized controlled trial. Clin Interv Aging. (2016) 11:9–16. doi: 10.2147/CIA.S96042 26766905 PMC4699540

[67] Phoemsapthawee J Ammawat W Leelayuwat N . The benefit of arm swing exercise on cognitive performance in older women with mild cognitive impairment. J Exercise Physiol Online. (2016) 19:123–36.

[68] Greblo Jurakic Z Krizanic V Sarabon N Markovic G . Effects of feedback-based balance and core resistance training vs. Pilates training on cognitive functions in older women with mild cognitive impairment: a pilot randomized controlled trial. Aging Clin Exp Res. (2017) 29:1295–8. doi: 10.1007/s40520-017-0740-9 28251569

[69] Kohanpour MA Peeri M Azarbayjani MA . The effects of aerobic exercise with lavender essence use on cognitive state and serum brain-derived neurotrophic factor levels in elderly with mild cognitive impairment. J HerbMed Pharmacol. (2017) 6:80–4.

[70] Lazarou I Parastatidis T Tsolaki A Gkioka M Karakostas A Douka S . International ballroom dancing against neurodegeneration: A randomized controlled trial in greek community-dwelling elders with mild cognitive impairment. Am J Alzheimers Dis Other Demen. (2017) 32:489–99. doi: 10.1177/1533317517725813 PMC10852896 28840742

[71] Morris JK Vidoni ED Johnson DK Van Sciver A Mahnken JD Honea RA . Aerobic exercise for Alzheimer’s disease: A randomized controlled pilot trial. PloS One. (2017) 12:e0170547. doi: 10.1371/journal.pone.0170547 28187125 PMC5302785

[72] Shimizu N Umemura T Matsunaga M Hirai T . Effects of movement music therapy with a percussion instrument on physical and frontal lobe function in older adults with mild cognitive impairment: a randomized controlled trial. Aging Ment Health. (2018) 22:1614–26. doi: 10.1080/13607863.2017.1379048 28937272

[73] Yoon DH Kang D Kim HJ Kim JS Song HS Song W . Effect of elastic band-based high-speed power training on cognitive function, physical performance and muscle strength in older women with mild cognitive impairment. Geriatr Gerontol Int. (2017) 17:765–72. doi: 10.1111/ggi.12784 27396580

[74] Doi T Verghese J Makizako H Tsutsumimoto K Hotta R Nakakubo S . Effects of cognitive leisure activity on cognition in mild cognitive impairment: results of a randomized controlled trial. J Am Med Dir Assoc. (2017) 18:686–91. doi: 10.1016/j.jamda.2017.02.013 28396179

[75] Choi W Lee S . Ground kayak paddling exercise improves postural balance, muscle performance, and cognitive function in older adults with mild cognitive impairment: A randomized controlled trial. Med Sci Monit. (2018) 24:3909–15. doi: 10.12659/MSM.908248 PMC6026380 29886507

[76] Donnezan LC Perrot A Belleville S Bloch F Kemoun G . Effects of simultaneous aerobic and cognitive training on executive functions, cardiovascular fitness and functional abilities in older adults with mild cognitive impairment. Ment Health And Phys Activity. (2018) 15:78–87. doi: 10.1016/j.mhpa.2018.06.001

[77] Hong SG Kim JH Jun TW . Effects of 12-week resistance exercise on electroencephalogram patterns and cognitive function in the elderly with mild cognitive impairment: A randomized controlled trial. Clin J Sport Med. (2018) 28:500–8. doi: 10.1097/JSM.0000000000000476 28727639

[78] Sungkarat S Boripuntakul S Kumfu S Lord SR Chattipakorn N . Tai chi improves cognition and plasma BDNF in older adults with mild cognitive impairment: A randomized controlled trial. Neurorehabil Neural Repair. (2018) 32:142–9. doi: 10.1177/1545968317753682 29353543

[79] Zhu Y Wu H Qi M Wang S Zhang Q Zhou L . Effects of a specially designed aerobic dance routine on mild cognitive impairment. Clin Interv Aging. (2018) 13:1691–700. doi: 10.2147/CIA PMC6138969 30237705

[80] Amjad I Toor H Niazi IK Afzal H Jochumsen M Shafique M . Therapeutic effects of aerobic exercise on EEG parameters and higher cognitive functions in mild cognitive impairment patients. Int J Neurosci. (2019) 129:551–62. doi: 10.1080/00207454.2018.1551894 30929591

[81] Bademli K Lok N Canbaz M Lok S . Effects of Physical Activity Program on cognitive function and sleep quality in elderly with mild cognitive impairment: A randomized controlled trial. Perspect Psychiatr Care. (2019) 55:401–8. doi: 10.1111/ppc.12324 30430592

[82] Mollinedo Cardalda I López A Cancela Carral JM . The effects of different types of physical exercise on physical and cognitive function in frail institutionalized older adults with mild to moderate cognitive impairment. A randomized controlled trial. Arch Gerontol Geriatr. (2019) 83:223–30. doi: 10.1016/j.archger.2019.05.003 31100545

[83] Choi W Lee S . The effects of virtual kayak paddling exercise on postural balance, muscle performance, and cognitive function in older adults with mild cognitive impairment: A randomized controlled trial. J Aging Phys Act. (2019) 27:861–70. doi: 10.1123/japa.2018-0020 31034303

[84] de Oliveira Silva F Ferreira JV Plácido J Sant’Anna P Araújo J Marinho V . Three months of multimodal training contributes to mobility and executive function in elderly individuals with mild cognitive impairment, but not in those with Alzheimer’s disease: A randomized controlled trial. Maturitas. (2019) 126:28–33. doi: 10.1016/j.maturitas.2019.04.217 31239114

[85] Fonte C Smania N Pedrinolla A Munari D Gandolfi M Picelli A . Comparison between physical and cognitive treatment in patients with MCI and Alzheimer’s disease. Aging (Albany NY). (2019) 11:3138–55. doi: 10.18632/aging.v11i10 PMC6555450 31127076

[86] Langoni CDS Resende TL Barcellos AB Cecchele B Knob MS Silva TDN . Effect of exercise on cognition, conditioning, muscle endurance, and balance in older adults with mild cognitive impairment: A randomized controlled trial. J Geriatr Phys Ther. (2019) 42:E15–e22. doi: 10.1519/JPT.0000000000000191 29738405

[87] Law LLF Mok VCT Yau MMK . Effects of functional tasks exercise on cognitive functions of older adults with mild cognitive impairment: a randomized controlled pilot trial. Alzheimers Res Ther. (2019) 11:98. doi: 10.1186/s13195-019-0548-2 31801630 PMC6894271

[88] Qi M Zhu Y Zhang L Wu T Wang J . The effect of aerobic dance intervention on brain spontaneous activity in older adults with mild cognitive impairment: A resting-state functional MRI study. Exp Ther Med. (2019) 17:715–22. doi: 10.3892/etm.2018.7006 PMC6307442 30651855

[89] Song D Yu DSF . Effects of a moderate-intensity aerobic exercise programme on the cognitive function and quality of life of community-dwelling elderly people with mild cognitive impairment: A randomised controlled trial. Int J Nurs Stud. (2019) 93:97–105. doi: 10.1016/j.ijnurstu.2019.02.019 30901716

[90] Tao J Liu J Chen X Xia R Li M Huang M . Mind-body exercise improves cognitive function and modulates the function and structure of the hippocampus and anterior cingulate cortex in patients with mild cognitive impairment. NeuroImage Clin. (2019) 23:101834. doi: 10.1016/j.nicl.2019.101834 31128522 PMC6535682

[91] Tarumi T Rossetti H Thomas BP Harris T Tseng BY Turner M . Exercise training in amnestic mild cognitive impairment: A one-year randomized controlled trial. J Alzheimers Dis. (2019) 71:421–33. doi: 10.3233/JAD-181175 31403944

[92] Bae S Lee S Lee S Jung S Makino K Harada K . The effect of a multicomponent intervention to promote community activity on cognitive function in older adults with mild cognitive impairment: A randomized controlled trial. Complement Ther Med. (2019) 42:164–9. doi: 10.1016/j.ctim.2018.11.011 30670238

[93] Wang L Wu B Tao H Chai N Zhao X Zhen X . Effects and mediating mechanisms of a structured limbs-exercise program on general cognitive function in older adults with mild cognitive impairment: A randomized controlled trial. Int J Nurs Stud. (2020) 110:103706. doi: 10.1016/j.ijnurstu.2020.103706 32739671

[94] Li L Liu M Zeng H Pan L . Multi-component exercise training improves the physical and cognitive function of the elderly with mild cognitive impairment: a six-month randomized controlled trial. Ann Palliat Med. (2021) 10:8919–29. doi: 10.21037/apm 34488379

[95] Khanthong P Sriyakul K Dechakhamphu A Krajarng A Kamalashiran C Tungsukruthai P . Traditional Thai exercise (Ruesi Dadton) for improving motor and cognitive functions in mild cognitive impairment: a randomized controlled trial. J Exerc Rehabil. (2021) 17:331–8. doi: 10.12965/jer.2142542.271 PMC8566108 34805022

[96] Yu AP Chin EC Yu DJ Fong DY Cheng CP Hu X . Tai Chi versus conventional exercise for improving cognitive function in older adults: a pilot randomized controlled trial. Sci Rep. (2022) 12:8868. doi: 10.1038/s41598-022-12526-5 35614144 PMC9131984

[97] Li F Harmer P Eckstrom E Fitzgerald K Winters-Stone K . Clinical effectiveness of cognitively enhanced tai ji quan training on global cognition and dual-task performance during walking in older adults with mild cognitive impairment or self-reported memory concerns: A randomized controlled trial. Ann Intern Med. (2023) 176:1498–507. doi: 10.7326/M23-1603 PMC13527859 37903365

[98] Parial LL Kor PPK Sumile EF Leung AYM . Dual-task zumba gold for improving the cognition of people with mild cognitive impairment: A pilot randomized controlled trial. Gerontologist. (2023) 63:1248–61. doi: 10.1093/geront/gnac081 35679826

[99] Uysal İ Başar S Aysel S Kalafat D Büyüksünnetçi A . Aerobic exercise and dual-task training combination is the best combination for improving cognitive status, mobility and physical performance in older adults with mild cognitive impairment. Aging Clin Exp Res. (2023) 35:271–81. doi: 10.1007/s40520-022-02321-7 36550323

[100] Zhang Q Zhu M Huang L Zhu M Liu X Zhou P . A study on the effect of traditional chinese exercise combined with rhythm training on the intervention of older adults with mild cognitive impairment. Am J Alzheimers Dis Other Demen. (2023) 38:15333175231190626. doi: 10.1177/15333175231190626 37489602 PMC10624104

[101] Cheng L Dong R Song C Li X Zhang L Shi M . Mediation effects of IL-1β and IL-18 on the association between vitamin D levels and mild cognitive impairment among Chinese older adults: A case-control study in Taiyuan, China. Front Aging Neurosci. (2022) 14:836311. doi: 10.3389/fnagi.2022.836311 35370605 PMC8966426

[102] Jadczak AD Makwana N Luscombe-Marsh N Visvanathan R Schultz TJ . Effectiveness of exercise interventions on physical function in community-dwelling frail older people: an umbrella review of systematic reviews. JBI Database System Rev Implement Rep. (2018) 16:752–75. doi: 10.11124/JBISRIR-2017-003551 29521871

[103] Cassilhas RC Lee KS Fernandes J Oliveira MGM Tufik S Meeusen R . Spatial memory is improved by aerobic and resistance exercise through divergent molecular mechanisms. Neuroscience. (2012) 202:309–17. doi: 10.1016/j.neuroscience.2011.11.029 22155655

[104] Torre MM Temprado JJ . A review of combined training studies in older adults according to a new categorization of conventional interventions. Front Aging Neurosci. (2021) 13:808539. doi: 10.3389/fnagi.2021.808539 35177975 PMC8844451

[105] Jindal A Ctori I Virgili G Lucenteforte E Lawrenson JG . Non-contact tests for identifying people at risk of primary angle closure glaucoma. Cochrane Database Syst Rev. (2020) 5:Cd012947. doi: 10.1002/14651858.CD012947.pub2 32468576 PMC7390269

[106] Sylwander C Sunesson E Andersson MLE Haglund E Larsson I . Experiences of health-promoting activities among individuals with knee pain: the halland osteoarthritis cohort. Int J Environ Res Public Health. (2022) 19(17):10529. doi: 10.3390/ijerph191710529 PMC9518063 36078245

[107] Ramírez-Vélez R Peña-Ibagon JC Martínez-Torres J Tordecilla-Sanders A Correa-Bautista JE Lobelo F . Handgrip strength cutoff for cardiometabolic risk index among Colombian children and adolescents: The FUPRECOL Study. Sci Rep. (2017) 7:42622. doi: 10.1038/srep42622 28195167 PMC5307343

[108] Thaker V Haagensen AL Carter B Fedorowicz Z Houston BW . Recombinant growth hormone therapy for cystic fibrosis in children and young adults. Cochrane Database Syst Rev. (2013) 6:Cd008901. doi: 10.1002/14651858.CD008901.pub2 23737090 PMC4465600

[109] Müller B Gaul C Glass Ä Reis O Jürgens TP Kropp P . Physical activity is associated with less analgesic use in women reporting headache-A cross-sectional study of the German migraine and headache society (DMKG). Pain Ther. (2022) 11:545–60. doi: 10.1007/s40122-022-00362-4 PMC9098765 35212968

[110] Xu D Yin Y Hou L Zhou H . A special acute care surgery model for dealing with dilemmas involved in emergency department in China. Sci Rep. (2021) 11:1723. doi: 10.1038/s41598-021-81347-9 33462376 PMC7813847

[111] Wang C . Sports-induced fatigue recovery of competitive aerobics athletes based on health monitoring. Comput Intell Neurosci. (2022) 2022:9542397. doi: 10.1155/2022/9542397 35602621 PMC9122678

[112] Niu A . EFFECT OF “TAI CHI” EXERCISE ON ANTIOXIDANT ENZYMES ACTIVITIES AND IMMUNITY FUNCTION IN MIDDLE-AGED PARTICIPANTS. Afr J Tradit Complement Altern Med. (2016) 13:87–90. doi: 10.21010/ajtcam.v13i5.12 28487898 PMC5416650

[113] Kang R Li Y Gao C Li J Zhang C Wang J . Discussion on repolarization reserve between patients with coronary heart disease and normal controls. Comput Math Methods Med. (2022) 2022:7944969. doi: 10.1155/2022/7944969 36035296 PMC9410869

[114] Sun H Soh KG Mohammadi A Wang X Bin Z Zhao Z . Effects of mental fatigue on technical performance in soccer players: A systematic review with a meta-analysis. Front Public Health. (2022) 10:922630. doi: 10.3389/fpubh.2022.922630 35937235 PMC9354787

[115] Duncan MJ Fowler N George O Joyce S Hankey J . Mental fatigue negatively influences manual dexterity and anticipation timing but not repeated high-intensity exercise performance in trained adults. Res Sports Med. (2015) 23:1–13. doi: 10.1080/15438627.2014.975811 25630242

[116] Takeuchi J Yanagimoto Y Sato Y Ochiai R Moriichi A Ishizaki Y . Efficacious interventions for improving the transition readiness of adolescents and young adult patients with chronic illness: A narrative review of randomized control trials assessed with the transition readiness assessment questionnaire. Front Pediatr. (2022) 10:983367. doi: 10.3389/fped.2022.983367 36245732 PMC9554476

[117] Latimer-Cheung AE Pilutti LA Hicks AL Martin Ginis KA Fenuta AM MacKibbon KA . Effects of exercise training on fitness, mobility, fatigue, and health-related quality of life among adults with multiple sclerosis: a systematic review to inform guideline development. Arch Phys Med Rehabil. (2013) 94:1800–28.e3. doi: 10.1016/j.apmr.2013.04.020 23669008

[118] Huang J Peng W Ding S Xiong S Liu Z . Fear of hypoglycemia and associated factors in hospitalized patients with type 2 diabetes: a cross−sectional study. Sci Rep. (2022) 12:20338. doi: 10.1038/s41598-022-24822-1 36434039 PMC9700846

[119] Meng Z Zhang J Shi J Zhao W Huang X Cheng L . Immunogenicity of influenza vaccine in elderly people: a systematic review and meta-analysis of randomized controlled trials, and its association with real-world effectiveness. Hum Vaccin Immunother. (2020) 16:2680–9. doi: 10.1080/21645515.2020.1747375 PMC7746244 32347787

[120] Ayati Z Yang G Ayati MH Emami SA Chang D . Saffron for mild cognitive impairment and dementia: a systematic review and meta-analysis of randomised clinical trials. BMC Complement Med Ther. (2020) 20:333. doi: 10.1186/s12906-020-03102-3 33167948 PMC7650148

[121] Barclay RE Stevenson TJ Poluha W Ripat J Nett C Srikesavan CS . Interventions for improving community ambulation in individuals with stroke. Cochrane Database Syst Rev. (2015) 2015:Cd010200. doi: 10.1002/14651858 25767912 PMC6465042

[122] Cyprien F Courtet P Maller J Meslin C Ritchie K Ancelin ML . Increased serum C-reactive protein and corpus callosum alterations in older adults. Aging Dis. (2019) 10:463–9. doi: 10.14336/AD.2018.0329 PMC6457060 31011488

[123] Zhang Y Li X Hu Y Yuan H Wu X Yang Y . Evaluation of mild cognitive impairment genetic susceptibility risks in a Chinese population. BMC Psychiatry. (2022) 22:93. doi: 10.1186/s12888-022-03756-y 35135506 PMC8822756

[124] Chang J Chen Y Liu C Yong L Yang M Zhu W . Effect of square dance exercise on older women with mild mental disorders. Front Psychiatry. (2021) 12:699778. doi: 10.3389/fpsyt.2021.699778 34393860 PMC8357995

[125] Yao L Fang H Leng W Li J Chang J . Effect of aerobic exercise on mental health in older adults: A meta-analysis of randomized controlled trials. Front Psychiatry. (2021) 12:748257. doi: 10.3389/fpsyt.2021.748257 34867538 PMC8634786

